# A rural-to-center artificial intelligence model for diagnosing Helicobacter pylori infection and premalignant gastric conditions using endoscopy images captured in routine practice

**DOI:** 10.1055/a-2721-6552

**Published:** 2025-11-26

**Authors:** Tsung-Hsien Chiang, Yen-Ning Hsu, Min-Han Chen, Yi-Ru Chen, Hsiu-Chi Cheng, Mei-Jin Chen, Fu-Jen Lee, Chi-Yang Chang, Chun-Chao Chang, Ming-Jong Bair, Jyh-Ming Liou, Chiuan-Jung Chen, Yen-Chung Chen, Hung Chiang, Chia-Tung Shun, Jui-Hsuan Liu, Han-Mo Chiu, Ming-Shiang Wu, Jiun-Yu Yu, Ruey-Shan Guo, Jaw-Town Lin, Yi-Chia Lee, Chu-Song Chen

**Affiliations:** 1 38006 Department of Integrated Diagnostics and Therapeutics, National Taiwan University Hospital, Taipei City, Taiwan; 2 Department of Internal Medicine, College of Medicine, National Taiwan University, Taipei City, Taiwan; 3 38006 Center of Intelligent Healthcare, National Taiwan University Hospital, Taipei City, Taiwan; 4 Department of Internal Medicine, National Cheng Kung University Hospital, College of Medicine, National Cheng Kung University, Tainan City, Taiwan; 5 Institute of Clinical Medicine, College of Medicine, National Cheng Kung University, Tainan City, Taiwan; 6 Institute of Molecular Medicine, College of Medicine, National Cheng Kung University, Tainan City, Taiwan; 7 Nangan Township, Bureau of Public Health, Matsu Islands, Lienchiang County, Taiwan; 8 485856 Department of Internal Medicine, Fu Jen Catholic University Hospital, New Taipei City, Taiwan; 9 School of Medicine, ollege of Medicine, Fu Jen Catholic University, New Taipei City, Taiwan; 10 Division of Gastroenterology and Hepatology, Department of Internal Medicine, Taipei Medical University Hospital, Taipei City, Taiwan; 11 Division of Gastroenterology and Hepatology, Department of Internal Medicine, School of Medicine, College of Medicine, Taipei Medical University, Taipei City, Taiwan; 12 Taipei Medical University Research Center for Digestive Medicine, Taipei Medical University, Taipei City, Taiwan; 13 Division of Gastroenterology, Department of Internal Medicine, Taitung Mackey Memorial Hospital, Taitung, Taiwan; 14 Mackay Medical College, New Taipei City, Taiwan; 15 Department of Medicine, National Taiwan University Cancer Center, Taipei City, Taiwan; 16 38006 Information Technology Office, National Taiwan University Hospital, Taipei City, Taiwan; 17 218818 Department of Pathology, National Yang Ming Chiao Tung University Hospital, Yilan City, Taiwan; 18 Taipei Institute of Pathology, Taipei City, Taiwan; 19 Department of Pathology, College of Medicine, National Taiwan University, Taipei City, Taiwan; 20 Lienchiang County Hospital, Nangan Township, Lienchiang County, Matsu Islands, Taiwan, Matsu Islands, Taiwan; 21 33561 Department and Graduate Institute of Business Administration, National Taiwan University, Taipei City, Taiwan; 22 Division of Gastroenterology and Hepatology, Department of Internal Medicine, E-Da Hospital, Kaohsiung City, Taiwan; 23 Department of Medical Research, National Taiwan University Hospital, Taipei City, Taiwan; 24 33561 Department of Computer Science and Information Engineering, National Taiwan University, Taipei City, Taiwan

## Abstract

**Background:**

Diagnosing *Helicobacter pylori* infection and premalignant gastric conditions typically requires ^13^ C urea breath testing or histological assessment, which are often unavailable in remote areas. A rural-to-center artificial intelligence (AI) model was developed and implemented to automatically evaluate upper endoscopy images from routine clinical practice.

**Methods:**

Endoscopic images were collected from a rural hospital on Matsu Islands and a tertiary center across Taiwan Strait. During model development (2020–2022), AI algorithms were trained, validated, and tested to exclude low-quality and non-gastric images, segment gastric regions, and enhance mucosal features for detecting *H. pylori* infection and premalignant conditions. During model implementation (2023–2024), endoscopic images from a rural hospital were transmitted to the medical center for AI analyses, with results promptly returned.

**Results:**

In the development phase, diagnostic accuracies were 92.8% (95%CI 88.9%–96.6%) for *H. pylori* , 88.6% (95%CI 87.2%–90.0%) for atrophic gastritis, and 88.0% (95%CI 86.5%–89.5%) for intestinal metaplasia. In the implementation phase, 3518 rural residents underwent ^13^ C urea breath testing or pepsinogen testing; 421 with positive results underwent endoscopy. No significant differences were observed between AI-predicted and clinically observed prevalence: *H. pylori* (13.9% vs. 12.9%; *P* = 0.55), atrophic gastritis (15.7% vs. 11.9%; *P* = 0.34), and intestinal metaplasia (27.6% vs. 22.4%; *P* = 0.32). Implementation-phase diagnostic accuracies were 91.3% (95%CI 88.0%–94.6%), 79.9% (95%CI 72.1%–86.3%), and 63.4% (95%CI 54.7%–71.6%), respectively.

**Conclusions:**

AI enabled physicians in resource-limited settings to rapidly assess gastric health using routinely captured endoscopic images, bridging gaps in access and expertise.

## Graphical Abstract

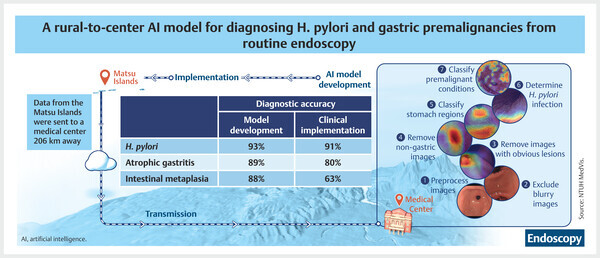

## Introduction

Gastric cancer remains a global health concern, with 968 784 new cases and 660 175 deaths reported in 2022, ranking fifth in both incidence and mortality 1 . The primary cause is *Helicobacter pylori* infection 2 , which can be treated with a short course of antibiotics to reduce cancer risk 3 4 . If left untreated, *H. pylori* can cause chronic inflammation and lead to premalignant gastric conditions that increase cancer risk. Individuals with these conditions may benefit from endoscopic surveillance for early cancer detection. However, such assessments are not routinely conducted due to time and resource demands, interobserver variability, specialist dependence, and difficulty determining biopsy number and sites 5 .

To reduce the threat of gastric cancer, primary and secondary prevention strategies can work together 6 . In recent years, a rapidly growing body of research has investigated the application of artificial intelligence (AI) in supporting the diagnosis of *H. pylori* infection and premalignant conditions, using either static images or real-time analyses with heatmaps or bounding box visualization (see **Table 1s** in the online-only Supplementary Material) 7 8 9 10 . However, in resource-limited settings, factors such as high clinical workloads, large image volumes, need for image enhancement, and limited computational infrastructure may place an additional burden on endoscopists. Utilizing routinely captured images to directly generate a global evaluation may offer a more efficient alternative. However, implementing such an approach may require a series of AI models capable of automatically preprocessing and selecting relevant images from the start-up to the replication of diagnostic reasoning of expert endoscopists and pathologists, a process that involves multiple steps rather than a single evaluation.

This novel concept was tested in a rural community on the Matsu Islands, located approximately 206 km across Taiwan Strait from Taiwan main island. This rural area, with limited health care resources, served as a pilot site for initiating *H. pylori* screening and eradication in 2004 11 12 , which was later extended using the colorectal cancer screening platform in 2014 4 , expanded to indigenous communities in 2018 13 , and ultimately scaled up to a population-wide implementation across Taiwan in 2026 14 . Although *H. pylori* screening and eradication have reduced the incidence of gastric cancer, cases may still occur among individuals who remain infected or have harbored premalignant conditions 15 . A cloud-based computation system was evaluated using a rural-to-center AI model to enable comprehensive yet efficient assessment of *H. pylori* infection and premalignant gastric conditions from endoscopic images captured during routine clinical practice.

## Methods

### Design of a rural-to-center AI model

**Fig. 1:**
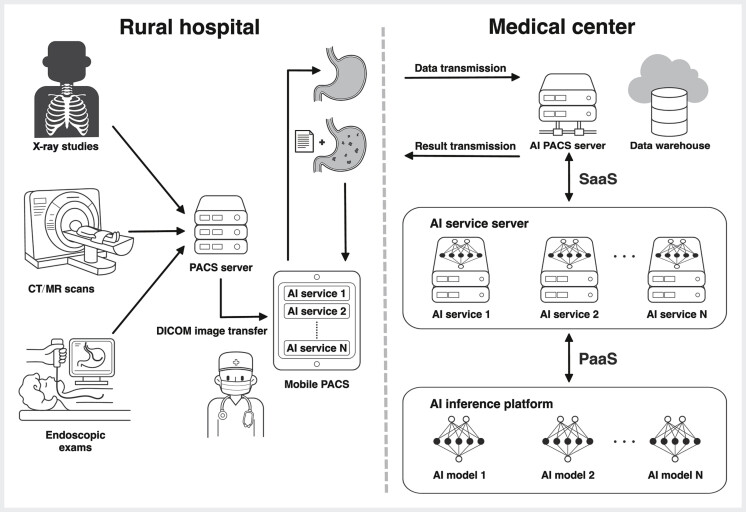
Infrastructure of a rural-to-center artificial intelligence (AI) model. The process begins with the transmission of endoscopic images in the digital imaging and communications in medicine (DICOM) format to a mobile picture archiving and communication system (PACS). The images are then sent to the cloud for computations using SaaS and PaaS technologies in the medical center. Finally, the AI-interpreted results are returned to the rural hospital for diagnosis. A web browser-based software, known as software as a service (SaaS), provides physicians with a user-friendly interface to select desired images and specify the types of AI inference required. The PACS server is upgraded to enable the transmission of selected images to the AI inference backend, which consists of deep learning models developed and managed by experts at a medical center, a service commonly referred to as platform as a service (PaaS). CT, computed tomography; MR, magnetic resonance. Source: NTUH MedVis.

The study involved both model development and implementation, using upper endoscopy images stored in the picture archiving and communication system (PACS) as input data. Clinical information, including ^13^ C urea breath testing and gastric histological evaluations, was used as the reference standard. During model development, AI models were trained, validated, and tested to exclude blurred and non-gastric images, segment gastric regions, and enhance mucosal details for diagnosing active *H. pylori* infection and premalignant conditions. During model implementation, upper endoscopy images from screening programs were transmitted from the frontend of a rural hospital to the backend of a medical center for computation. The AI-generated results were immediately returned to the rural hospital via a mobile PACS platform (EBM Technologies Inc., Taipei, Taiwan) ( Fig. 1 ). An online video demonstration shows how the system functions (https://youtu.be/bLOS1rBJLUw). The study followed the quality assessment of AI preclinical studies in diagnostic endoscopy (QUAIDE) checklist 16 .

### Data acquisition

Data were collected from two sources: a rural hospital (Lienchiang County Hospital, Matsu Islands) and a medical center (National Taiwan University Hospital, Taipei). **Fig. 1s** illustrates the geographic locations. The medical center’s centralized data warehouse contained electronic medical records, including chart records, laboratory data, examination reports, pathological results, and medical images, gathered from 10 affiliated hospitals 17 . Data for model training and validation were primarily randomly selected from this warehouse. Data from the rural hospital were mainly used for validation and testing.

### Model development

**Fig. 2:**
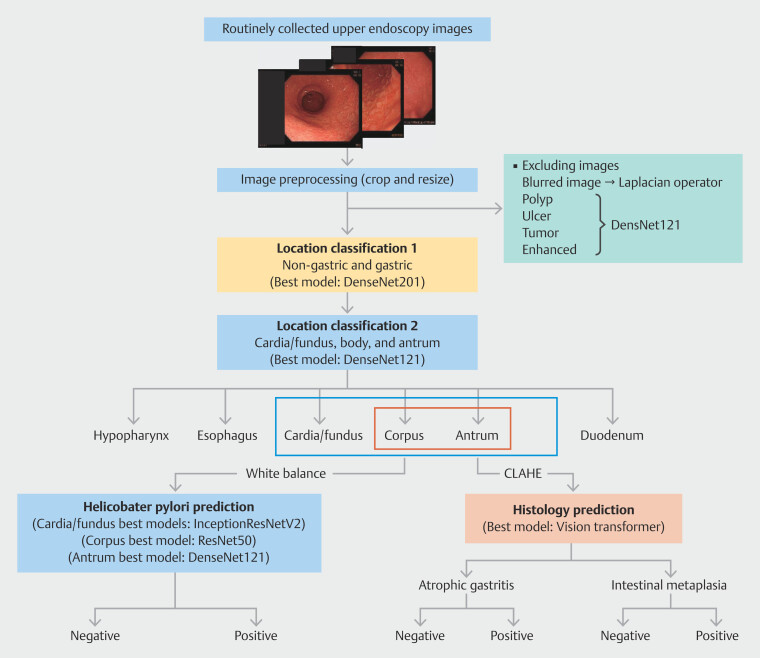
Structure of the artificial intelligence models. CLAHE, contrast-limited adaptive histogram equalization.

**Fig. 3:**
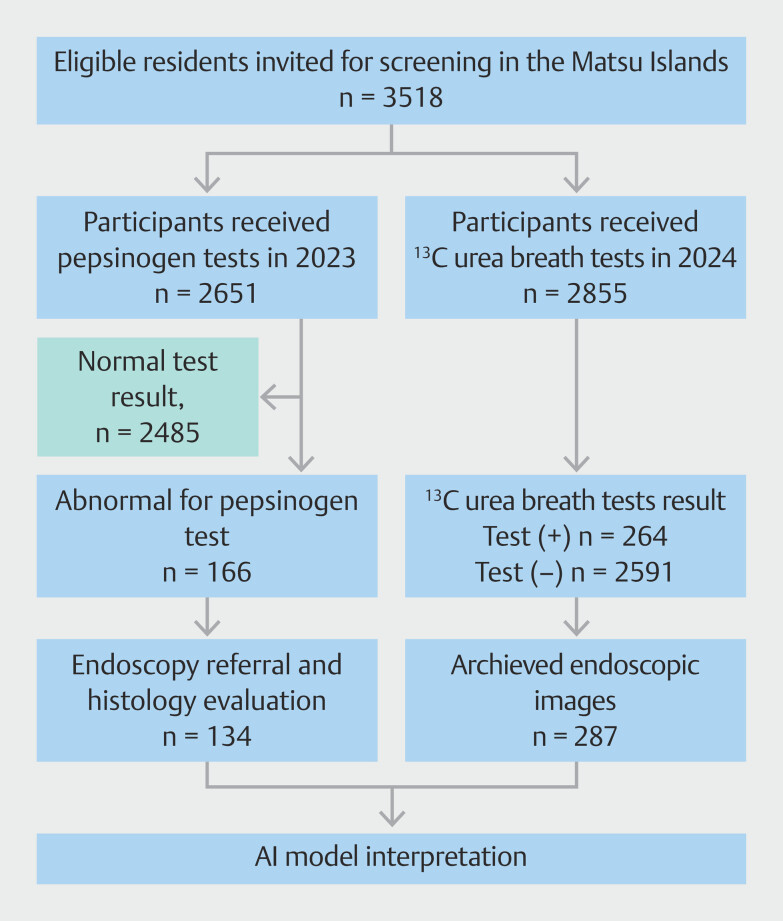
Flow chart of the community-based screening program implemented in the Matsu Islands, Taiwan. An abnormal result for pepsinogen (PG) testing was based on the cutoff values of PG I <30 μg/L or PG I/II ratio <3.

**Fig. 4:**
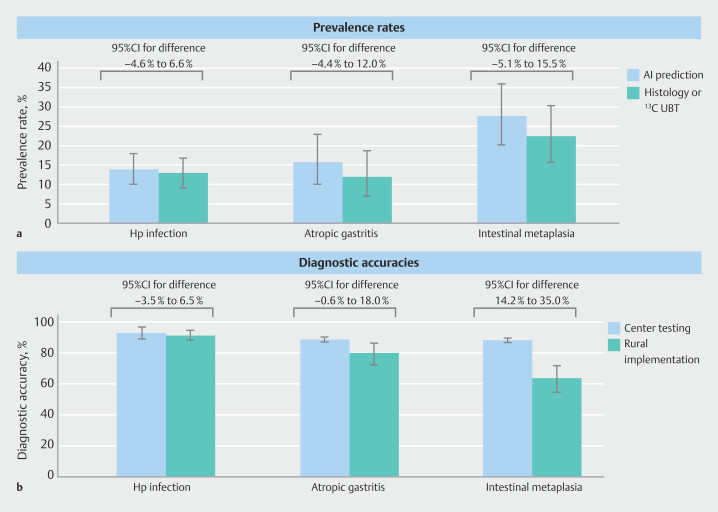
Comparison of predictions made by the artificial intelligence models with observations based on endoscopic biopsy histology evaluation and ^13^ C urea breath testing in the rural community-based screening programs implemented in the Matsu Islands, Taiwan. **a** Prevalence rates. **b** Diagnostic accuracies. AI, artificial intelligence; Hp, *Helicobacter pylori* ; UBT, urea breath test.

The model development process was designed to mirror the diagnostic workflow of expert endoscopists and pathologists ( Fig. 2 ). The upper endoscopy examination was conducted in accordance with the systematic screening protocol 18 . Each endoscopic image was selected and classified by experienced endoscopists (Drs. Chiang TH and Lee YC), each with over 20 years of experience, based on the purpose and desired outcome of each step. Prior to machine learning, the images were preprocessed to remove irrelevant elements ( **Fig. 2s** ). The Laplacian method was used to remove blurred images, with a threshold set at a Laplacian score of 800 ( **Fig. 3s** ) 19 . Images were divided into training, validation, and testing datasets. Each image was further segmented into patches, which were classified based on the proportion of patches diagnosed with a specific outcome, following the general principle of convolutional neural networks ( **Supplementary methods** ). A cutoff value, determined during the training phases, was used to assign image-level diagnoses. The validation process incorporated an iterative upgrade module that analyzed misclassified cases to enable continuous model optimization. Model performance was subsequently evaluated using the testing dataset. The development workflow is summarized in Table 1 , with additional details provided in **Fig. 4s** , which demonstrates this process using the histological model as an example. The process is described step by step below.

**Table:** **Table 1** Data for the model training, validation, and testing, and the model selection during model development.

System development	Purpose ^1^			Model selection	Selected model	Outcomes
	Training	Validation	Testing			
Step 1 Exclude organic lesions and enhanced images	Center: 70% Images: 2449 Normal: 605 Polyp: 448 Tumor: 454 Ulcer: 513 Enhanced: 429	Center: 20% Images: 699 Normal: 173 Polyp: 128 Tumor: 130 Ulcer: 146 Enhanced: 122	Center: 10% Images: 350 Normal: 87 Polyp: 64 Tumor: 65 Ulcer: 73 Enhanced: 61	ResNet50 InceptionV3 InceptionResNetV2 Xception DenseNet121 MobileNetV2 EfficientNetV2B0	DenseNet121	Organic lesions and enhanced images: 0 Normal image: 1
Step 2 Classify the stomach from non-stomach images	Center: 70% Patients: 1971 Images: 3783 Stomach: 1952 Non-stomach: 1831	Center: 20% Patients: 577 Images: 1135 Stomach: 598 Non-stomach: 537	Center: 10% Patients: 244 Images: 487 Stomach: 263 Non-stomach: 224	ResNet50 DenseNet121 DenseNet201 Xception InceptionV3 InceptionResNetV2	DenseNet201	Non-stomach: 0 Stomach: 1
Step 3 Classify stomach images according to the anatomical locations	Center: 80% Patients: 940 Images: 12 803 Cardia/fundus: 1248 Corpus: 6566 Antrum: 4989	Center: 20% Patients: 235 Images: 3381 Cardia/fundus: 336 Corpus: 1734 Antrum: 1131	Rural: 100% Patients: 561 Images: 5541 Cardia/fundus: 508 Corpus: 2287 Antrum: 2746	ResNet50 DenseNet121 DenseNet201 Xception InceptionV3 InceptionResNetV2	DenseNet121	Cardia/fundus: 0 Corpus: 1 Antrum: 2
Step 4 Classify the presence of active *H. pylori* infection	Center: 70% Patients: 1466 Images: 8978 Cardia/fundus: 1134 Corpus: 3969 Antrum: 3875	Center: 20% Patients: 360 Images: 2362 Cardia/fundus: 298 Corpus: 1047 Antrum: 1017	Center: 10% Patients: 178 Images: 1204 Cardia/fundus: 152 Corpus: 532 Antrum: 520	ResNet50 DenseNet121 InceptionV3 InceptionResNetV2 EfficientNet-B0 EfficientNet-B1	InceptionResNetV2 (cardia/fundus) ResNet50 (corpus) DenseNet121 (antrum)	*H. pylori* (–): 0 *H. pylori* (+): 1
Step 5 Classify the presence of premalignant gastric conditions	Center: 100% Rural: 70% Patients: 1493 Images: 17 178 Corpus: 9437 Antrum: 7741	Rural: 10% Patients: 56 Images: 476 Corpus: 249 Antrum: 227	Rural: 20% Patients: 97 Images: 853 Corpus: 388 Antrum: 465	VGG11 VGG19 ResNet50 ResNet152 DenseNet121 DenseNet201 EfficientNet-B0 EfficientNet-B4 EfficientNet-B6 Vision Transformer	Vision Transformer	No premalignant condition: 0 With premalignant condition: 1
^1^ Center = National Taiwan University Hospital, a medical center; rural hospital = Lienchiang County Hospital on the Matsu Islands, a district hospital on an offshore island.						

#### Step 1: Remove organic lesions and enhanced images

The study focused on background gastric mucosa. Images of visible organic lesions, such as tumors, polyps, and ulcers, were excluded, as these would already prompt clinical management and were not within the intended focus of this study. Enhanced images, such as narrow-band images, were also excluded because of different interpretations ( **Fig. 5s** ).

#### Step 2: Remove non-gastric images

Upper endoscopy examinations also included the hypopharynx, esophagus, and duodenum, while the related images were irrelevant to the study’s purpose ( **Fig. 6s** ).

#### Step 3: Classify different regions of the stomach

Different anatomical locations of the stomach may show distinct mucosal manifestations for both *H. pylori* infection and premalignant conditions. Additionally, the prospective histological assessment was limited to the corpus and antrum. Therefore, it was essential to differentiate between these anatomical locations ( **Fig. 7s** ).

#### Step 4: Classify the presence of active *H. pylori* infection

As the population of Matsu Islands underwent previous mass eradication of *H. pylori* 14 , data to evaluate the *H. pylori* infection were obtained from the medical center. The prevalence rate of active *H. pylori* infection was found to be 55.9% using ^13^ C urea breath testing as the reference standard. This step involved three AI models for the three different locations (antrum, corpus, cardia/fundus), as they may show different mucosal patterns of *H. pylori* infection ( **Fig. 8s** ) 20 . Endoscopists (Drs. Chiang TH and Lee YC) reviewed and selected both infected and non-infected images to match the corresponding breath test results. The classification process had to start with image enhancement using white balance adjustments to achieve sufficient model performance ( **Fig. 9s** ).

#### Step 5: Classify the presence of premalignant gastric conditions

Histological diagnoses of premalignant gastric conditions were not routinely available in standard upper endoscopy practice. In the rural hospital, these data were prospectively collected from a population-based *H. pylori* screen-and-treat program on the Matsu Islands 11 12 . At the medical center, these data were collected as part of randomized clinical trials 21 22 . The prevalence and severity of premalignant conditions using the modified Sydney protocol are shown in **Table 2s** . In brief, gastric mucosa biopsy specimens were obtained from the antrum (2–3 cm from the pylorus along the greater and lesser curvatures) and corpus (one each from the lesser and greater curvatures at the middle corpus) 23 . Senior histopathologists, unaware of participants’ clinical status, performed all histological assessments (Drs. Chen YC, Chiang H, and Shun CT). The specimens were graded as acute inflammation (polymorphonuclear infiltrates), chronic inflammation (lymphoplasmacytic infiltrates), atrophic gastritis (loss of glandular tissue and fibrous replacement), or intestinal metaplasia (presence of goblet cells and absorptive cells). The severity of each category was rated as none, mild, moderate, or marked, enabling classification of the severity of premalignant conditions using the Operative Link for Gastritis Assessment of Atrophic Gastritis (OLGA) and Operative Link for Gastritis Assessment of Intestinal Metaplasia (OLGIM) criteria, ranging from stage 0 to stage 4 24 25 . The weighted kappa values for gastric atrophy and intestinal metaplasia were 0.62 and 0.74, respectively, among the pathologists 11 12 . The histological diagnoses served as the reference standard. As high-stage diseases were rare, the classification was dichotomized based on the presence or absence of premalignant conditions in the antrum and corpus (stages 0 and ≥1). Given the known patchy distribution of premalignant conditions, stored images were reviewed and selected by endoscopists (Drs. Chiang TH and Lee YC) to ensure alignment with corresponding histological diagnoses. To achieve sufficient model performance, this step began with image enhancement using contrast-limited adaptive histogram equalization 26 , following the principles of image-enhanced endoscopy ( **Fig. 10s** ).

### A per-patient assessment

For implementation, analyses needed to convert the per-image basis to a per-patient diagnosis for both *H. pylori* infection and premalignant conditions. As images captured from the same anatomical location (antrum, corpus, or fundus/cardia) could yield varying interpretations, some indicating *H. pylori* infection or premalignant changes and others not, a voting procedure was applied. A patient was classified as having a specific outcome if the percentage of positive images among all gastric images exceeded a predefined cutoff. This cutoff was calibrated to align with the observed prevalence rate 11 12 17 21 22 .

### Interpretability analyses

Given that the AI model may function as a black box, interpretability analyses were conducted using two complementary approaches to ensure transparency and clinical relevance, including the per-image approach and the comprehensive approach. First, gradient-weighted class activation mapping was applied to generate heatmaps for each image, highlighting regions of model focus. This enabled case-by-case evaluation of whether the AI attention aligned with clinical judgment at each diagnostic step, as visualized through the mobile PACS ( **Fig. 11s** ).

Second, a comprehensive interpretability analysis was performed by assessing whether the model high-attention classifications corresponded with established associations among *H. pylori* infection, premalignant conditions, and gastric cancer. Raw endoscopic images were obtained from an independent dataset comprising patients diagnosed with gastric cancer, identified through a data warehouse linked to the Taiwan Cancer Registry (2004–2022). Cases included patients who had undergone upper endoscopy at least 180 days prior to their cancer diagnosis and had archived images, representing individuals with either undetected gastric cancer or a precancerous condition. Controls were randomly selected from patients without a gastric cancer diagnosis during the same period. Archived endoscopic images from both groups were processed through steps 1 to 5 of the AI models. Classification results for the cancer and non-cancer groups were then analyzed to determine whether the AI interpretability aligned with established clinical risk factors. The results were assessed using Shapley additive explanations values, which measured the change in model predictability when the risk factor was present versus absent 27 .

### Model implementation

Based on the population registry, a community-based screening program invited residents of the Matsu Islands aged 30 years or older to undergo ^13^ C urea breath testing or pepsinogen testing on a biennial basis in alternating years 11 12 15 . Individuals who tested positive for active *H. pylori* infection were referred for eradication therapy, while those with abnormal pepsinogen results were referred for upper endoscopy examination and histological evaluation according to the random biopsy protocol, as detailed above (Sydney protocol) 23 . Routinely captured upper endoscopy images from participants were transmitted to the AI inference backend at the medical center, and the AI-generated results were instantly relayed back to the rural hospital.

### Ethical approval

The study was approved by the Ethics Committee of National Taiwan University Hospital (No. 201402061RINA), and all participants provided written informed consent.

### Statistical analyses

Patients’ baseline characteristics were summarized as percentages for categorical variables and as means with SD for continuous variables. During model development, the discriminative performance of the AI models was assessed using sensitivity, specificity, and diagnostic accuracy, with the corresponding 95%CIs to evaluate statistical significance. The model with the highest accuracy at each step was selected and integrated into the system. During the implementation phase, the McNemar’s test was used to compare the prevalence rates between AI-predicted and observed outcomes in paired data from the same rural participants. Diagnostic accuracies were compared with those from the development phase using a two-sample proportion test between two independent populations from rural and center hospitals. As the study was exploratory, adjustments for multiple comparisons were not applied to avoid potential false-negative findings, aligning with the goal of generating new insights.

For computation, Python within the TensorFlow 2.8 framework was used on the NVIDIA DGX A100 GPU (40G; NVIDIA Corporation, Santa Clara, California, USA). A 2-sided *P* value of <0.05 was considered statistically significant for all outcomes.

### Cost-effectiveness analysis

The AI-assisted approach may also increase the medical burden of *H. pylori* testing and endoscopic surveillance, as it generates additional information that may prompt further work-up. A cost-effectiveness analysis was conducted with the primary end point of life-years gained, estimated by translating screening-related mortality reductions into life-years gained ( **Supplementary methods** ). The structure of the Markov model and the data inputs are presented in **Fig. 12s** , **Fig. 13s** , and **Table 3s** . The incremental cost-effectiveness ratio was calculated as the difference in costs divided by the difference in life-years between the AI-assisted strategy and routine practice.

The analyses were performed using TreeAge Pro 2024 (TreeAge Software, Inc., Williamstown, Massachusetts, USA).

## Results

### Model development

The best-performing models for each step are shown in Table 1 . Details of the model selection process are provided in **Fig. 14s** . In step 1, all seven deep learning models demonstrated strong performance. The best-performing model, based on DenseNet121, achieved sensitivity, specificity, and accuracy of 95.1% (95%CI 92.3%–97.9%), 91.2% (95%CI 88.3%–94.1%), and 97.0% (95%CI 94.8%–99.2%), respectively, on the testing set for excluding organic lesions and enhanced images ( Table 2 ). For step 2, all six models showed excellent performance, with the best-performing DenseNet201 model achieving sensitivity, specificity, and accuracy of 99.6% (95%CI 99.2%–100%), 100%, and 99.9% (95%CI 99.9%–100%), respectively, for excluding non-stomach images. For step 3, the performance was slightly lower compared with the first two steps, as the cardia/fundus, corpus, and antrum are continuous structures and there were borderline areas. The best-performing DenseNet121 model achieved sensitivity, specificity, and accuracy values of 90.5% (95%CI 89.5%–91.5%), 95.0% (95%CI 94.6%–95.4%), and 98.2% (95%CI 98.0%–98.4%), respectively, for differentiating between these three locations.

**Table:** **Table 2** Model development based on the best-performing model according to the sensitivity, specificity, and accuracy in the testing set.

Steps	Sensitivity (95%CI), %	Specificity (95%CI), %	Accuracy (95%CI), %
Step 1: Exclude organic lesions and enhanced images	95.1 (92.3–97.9)	91.2 (88.3–94.1)	97.0 (94.8–99.2)
Step 2: Classify the stomach from non-stomach images	99.6 (99.2–100)	100	99.9 (99.9–100)
Step 3: Classify stomach images according to anatomical location			
Cardia/fundus	74.6 (72.7–76.5)	97.9 (97.7–98.1)	98.0 (97.1–98.9)
Corpus	90.6 (90.0–91.2)	91.1 (90.6–91.6)	97.0 (96.5–97.5)
Antrum	93.4 (92.9–93.9)	94.6 (94.2–95.0)	98.7 (98.4–99.0)
Overall	90.5 (89.5–91.5)	95.0 (94.6–95.4)	98.2 (98.0–98.4)
Step 4: Classify the presence of active *H. pylori* infection	95.0 (92.9–97.2)	91.2 (85.6–96.6)	92.8 (88.9–96.6)
Step 5: Classify the presence of premalignant gastric conditions			
Atrophic gastritis	79.4 (75.3–83.5)	74.7 (72.8–76.6)	88.6 (87.2–90.0)
Intestinal metaplasia	78.2 (74.7–81.7)	71.3 (69.1–73.5)	88.0 (86.5–89.5)

For step 4, using InceptionResNetV2 for the cardia/fundus, ResNet50 for the corpus, and DenseNet121 for the antrum, the overall sensitivity, specificity, and accuracy were 95.0% (95%CI 92.9%–97.2%), 91.2% (95%CI 85.6%–96.6%), and 92.8% (95%CI 88.9%–96.6%), respectively, for detecting active *H. pylori* infection. For step 5, the best-performing Vision Transformer model achieved sensitivity, specificity, and accuracy of 79.4% (95%CI 75.3%–83.5%), 74.7% (95%CI 72.8%–76.6%), and 88.6% (95%CI 87.2%–90.0%), respectively, for differentiating atrophic from non-atrophic gastric mucosae. The same Vision Transformer model achieved sensitivity, specificity, and accuracy of 78.2% (95%CI 74.7%–81.7%), 71.3% (95%CI 69.1%–73.5%), and 88.0% (95%CI 86.5%–89.5%), respectively, for differentiating between the presence and absence of intestinal metaplasia.

### The per-patient assessment

For the per-patient assessment, the diagnosis of active *H. pylori* infection was determined by a majority vote (i.e. when the number of positive images divided by the total number of images was ≥50%). Atrophic gastritis was considered positive when the proportion of positive images exceeded 20%. Intestinal metaplasia was considered positive when the proportion of positive images exceeded 7%.

### Interpretability analysis

For the per-image approach, gradient-weighted class activation mapping generated heatmaps for the regions of model focus for the representative images ( **Figs. 5s–7s** , **9s–11s** ), and these generally aligned with clinical judgement. For the comprehensive approach, a total of 326 patients (35 670 images) with subsequent gastric cancer and 6369 patients (168 116 images) without subsequent gastric cancer were enrolled. The mean time between gastric cancer diagnosis and the last upper endoscopy was 4.1 years (SD 3.5). The raw upper endoscopy images were preprocessed and analyzed by AI through steps 1 to 5, using the best-performing model selected for each step. The interpretation was finally transformed to a per-patient basis for the diagnosis of active *H. pylori* infection and premalignant conditions. Two AI models, logistic regression model, and support vector machine, demonstrated consistent results on the testing sets ( **Table 4s** ), with sensitivity, specificity, and accuracy of 89.8% (95%CI 83.9%–95.7%), 81.6% (95%CI 74.3%–88.9%), and 90.2% (95%CI 84.4%–96.0%), respectively, which were significantly better than those of the model solely based on age and sex, with sensitivity, specificity, and accuracy of 75.5% (95%CI 67.5%–83.5%), 59.4% (95%CI 50.9%–68.0%), and 73.0% (95%CI 64.8%–81.2%), respectively (all *P* < 0.001). Evaluation of variable importance indicated that active *H. pylori* infection and the presence of intestinal metaplasia, as generated by the AI models, were highly influential ( **Fig. 15s** ).

### Model implementation

Between March 3, 2023, and April 30, 2024, a community-based screening program was conducted on the Matsu Islands. Of the 3518 eligible individuals aged 30 years or older who were invited, 2651 (mean age 54.0 years [SD 13.4]) participated in pepsinogen testing in 2023, while 2855 (mean age 54.4 years [SD 13.6]) underwent ¹³C urea breath testing in 2024 ( **Table 5s** ). A total of 166 individuals (6.3%) tested positive for pepsinogen, and 264 individuals (9.2%) tested positive for *H. pylori* infection ( Fig. 3 ). Among the pepsinogen-positive individuals, 134 underwent endoscopic examination and histological evaluation. Additionally, 287 individuals who received *H. pylori* testing had upper endoscopy images stored in the rural hospital PACS.

As shown in Fig. 4 , AI-predicted vs. observed prevalence rates were: *H. pylori* (13.9% vs. 12.9%; *P* = 0.55), atrophic gastritis (15.7% vs. 11.9%; *P* = 0.34), and intestinal metaplasia (27.6% vs. 22.4%; *P* = 0.32), at an individual patient level. No significant differences were observed across all comparisons. Implementation-phase diagnostic accuracies were 91.3% (95%CI 88.0%–94.6%) for *H. pylori* , 79.9% (95%CI 72.1%–86.3%) for atrophic gastritis, and 63.4% (95%CI 54.7%–71.6%) for intestinal metaplasia. Compared with the development-phase accuracies, the results were not significantly different for *H. pylori* infection and atrophic gastritis, whereas a significant difference was shown for intestinal metaplasia. In the community, five individuals with a history of gastric cancer had endoscopic imaging archived in the PACS at the rural hospital, prior to gastric cancer diagnosis. The AI model diagnosed *H. pylori* infection and intestinal metaplasia in all cases, while atrophic gastritis was identified in one.

### Cost-effectiveness analysis

An incremental gain of 2.02 life-years and a cost reduction of USD 93.7 were observed with the AI-assisted strategy compared with routine practice ( **Fig. 16s** ), resulting in an incremental cost-effectiveness ratio of –46.4 and a cost-saving result. The benefits of *H. pylori* eradication and early gastric cancer detection triggered by AI may outweigh the burden associated with advanced cancer treatments and reduced life expectancy under the traditional approach.

## Discussion

This study presents a novel AI-based approach that helps diagnose *H. pylori* infection and premalignant gastric conditions using routinely captured images. Unlike traditional methods with longer turnaround times, it delivers results within minutes. Validated in both well-resourced and underserved settings, it offers a practical tool to support frontline physicians by bridging expertise gaps and geographic barriers.

Previous AI-based studies have shown promising results, with a pooled accuracy of 80%–96% for diagnosing active *H. pylori* infection and 90%–96% for diagnosing premalignant gastric conditions 7 8 9 10 . Previous studies mainly used highly selected images from well-resourced settings and focused on the proof-of-concept stage. In contrast, this study integrated a series of AI models in an end-to-end manner to simulate the full diagnostic process, offering several unique insights. First, blurred endoscopic images must be excluded from stepwise classification to avoid misdiagnosis. Second, due to the varied mucosal patterns of *H. pylori* -related gastritis and premalignant lesions across different stomach regions, images must be segmented by region to enable accurate classification. Third, image enhancement is necessary in order to reveal mucosal details, enabling reliable differentiation between active infection and premalignant conditions. Fourth, a per-patient assessment based on a voting strategy efficiently correlates with gastric cancer risk and is practical for first-line application.

Virtual chromoendoscopy staging scores, such as the Endoscopic Grading of Gastric Intestinal Metaplasia (EGGIM) 28 , which assesses intestinal metaplasia using narrow-band imaging or linked color imaging, can enhance interpretation and have been shown to be applicable with AI 29 . However, routine application of EGGIM may be challenging for less experienced endoscopists, particularly among general practitioners outside specialized centers who primarily focus on detecting organic lesions in the esophagus, stomach, and duodenum based on clinical symptoms. Although the rapid urease test can accurately detect *H. pylori* when infection is suspected, some cases may still be missed because evaluation of normal-appearing gastric mucosa is often overlooked in routine practice. AI has the potential to extract additional information from standard endoscopic images and prompt appropriate clinical management.

This study’s strength is its efficient end-to-end approach, from meticulous data collection through model development to real-world evaluation. Generalizability was shown through training, validation, and testing, and implementation in both a large medical center and a rural hospital. However, several limitations should be acknowledged. First, *H. pylori* assessment and histological grading, tasks requiring the identification of subtle mucosal and vascular patterns, were sensitive to image resolution. Although self-attention-based image enhancement improved interpretability, further robustness could be achieved using higher-resolution images and optimized lighting conditions when the images are taken. Virtual chromoendoscopy-based AI interpretation is a valuable endeavor but requires a higher level of expert annotation and sufficient training data 29 . Second, images not meeting quality standards were excluded, which may render some cases uninterpretable. However, this limitation underscores the model’s potential to drive quality improvement in upper endoscopic imaging. Third, the AI system provided histological predictions for all antrum and body images, far exceeding the limited sampling in the OLGA and OLGIM biopsy protocols 24 25 . While AI-predicted and observed prevalence rates for *H. pylori* infection and premalignant conditions were similar during the implementation phase, the accuracy for detecting intestinal metaplasia was lower than that in the development phase, where AI models were trained on selected images (akin to targeted biopsies). In contrast, unselected images were directly input into the system during implementation. The reduced accuracy was attributed to the complexity of the multistep pipeline, which may increase the risk of misclassification; incorporating an interactive module could help identify and correct erroneous cases. It was also related to the patchy distribution of intestinal metaplasia, unlike the more diffuse changes seen in atrophic gastritis and *H. pylori* infection, particularly when biopsies were taken from normal-appearing mucosae under a standardized protocol. Fourth, the screening program followed organized screening principles; thus, only a subset of individuals with positive noninvasive tests were eligible for endoscopy. The favorable cost-effectiveness results relied on assumptions regarding the magnitude of gastric cancer prevention and early gastric cancer detection. Continued cohort follow-up over a longer period is needed to further support this hypothesis. Fifth, real-time AI analyses, similarly to polyp detection in colonoscopy, hold promise for enhancing diagnostic accuracy during upper endoscopy but are still in early development and mainly focused on organic lesion detection 30 31 32 33 . As shown in the image heatmaps, diffuse mucosal changes and the patchy nature of premalignant lesions complicated real-time interpretation and increased the workload of semantic segmentation. A global assessment based on routinely archived images may offer a simpler and more efficient alternative. While the research advances early screening capabilities, challenges remain in interpreting AI-generated results. Since current clinical guidelines are based on the diagnostic expertise of human physicians developed over years of experience, it is crucial to evaluate whether AI outputs align with these standards, which may require the development of new follow-up and treatment plans. Sixth, the low proportion and case numbers limited the ability to categorize lesions using the original OLGA/OLGIM system, in which advanced stages requiring surveillance are primarily defined as stage III–IV, according to MAPS III 34 and American College of Gastroenterology 35 guidelines. Nonetheless, in the interpretability analyses, this dichotomization remained consistent with established risk levels for premalignant conditions, particularly intestinal metaplasia.

In conclusion, this study demonstrates that step-by-step AI models can automatically extract valuable insights from routinely captured upper endoscopy images to evaluate *H. pylori* infection and premalignant gastric conditions, providing a novel approach to extending AI technologies to rural areas and reducing disparities in stomach health management.

## Supplementary Material

Supplementary Material Zusatzmaterial
