# Supplementary material for: A rural-to-center artificial intelligence model for diagnosing Helicobacter pylori infection and premalignant gastric conditions using endoscopy images captured in routine practice
Source: Endoscopy. 2025 Nov 26;58(4):343–54. doi: 10.1055/a-2721-6552 (PMC13063403; doi:10.1055/a-2721-6552)
Supplement: Supplementary file 1 — Supplementary Material [file 10-1055-a-2721-6552_27408773.pdf]

APPENDIX

Tsung-Hsien Chiang, Yen-Ning Hsu, Min-Han Chen, Yi-Ru Chen, Hsiu-Chi Cheng, Mei-Jin Chen, Fu-Jen Lee, Chi-Yang Chang, Chun-Chao Chang, Ming-Jong Bair, Jyh-Ming Liou, Chiuan-Jung Chen, Yen-Chung Chen, Hung Chiang, Chia-Tung Shun, Jui-Hsuan Liu, Han-Mo Chiu, Ming-Shiang Wu, Jiun-Yu Yu, Ruey-Shan Guo, Jaw-Town Lin, Yi-Chia Lee, MD, Chu-Song Chen

A Rural-to-Center Artificial Intelligence Model for Diagnosing *Helicobacter pylori* Infection and Premalignant Gastric Conditions Using Endoscopy Images Captured in Routine Practice

Contents

Supplementary methods 3
General machine learning process 3
Cost-effectiveness analysis 3
Natural course model 3
Decision analysis 4
Markov cycles 4
Data input 4
Assumptions 4
End points 5
Supplementary results 5
Table 1s Recent studies for the use of artificial intelligence to assist in diagnosing *Helicobacter pylori* infection and premalignant conditions using static images or real-time analyses. 6
Table 2s Baseline characteristics of patients with intragastric histological results for model development. 9
Table 3s Data for the cost-effectiveness analysis. 10
Table 4s Comprehensive interpretation analyses in gastric cancer risk evaluation, including the age, sex, and AI assisted diagnoses of active *H. pylori* infection and premalignant conditions, based on the logistic regression model. 12
Table 5s Baseline characteristics of the participants for the screening programs in the model implementation phase. 13
Fig. 1s The geographical distance between the Matsu Islands and Taiwan. 14
Fig. 2s Preprocessing of upper endoscopic images. 15
Fig. 3s Exclusion of the blurred images. 16
Fig. 4s Rationale for the artificial intelligence models using the histological prediction model as an example. 17
Fig. 5s Exclusion of enhanced images and organic lesions. 18
Fig. 6s Classification of upper endoscopy images according to the non-stomach images and stomach images. 19

Fig. 7s Classification of stomach images according to the antrum, corpus, and cardia/fundus. 20
Fig. 8s Mucosal patterns of *H. pylori* infection according to the anatomical locations. 21
Fig. 9s Classification of stomach images according to the presence of *H. pylori* infection. 22
Fig. 10s Classification of stomach images according to the presence of premalignant gastric conditions. 24
Fig. 11s Per-image basis interpretability analysis, as visualized through the mobile PACS. 26
Fig. 12s Strategies and health status in the decision tree model.. 27
Fig. 13s Examples of pathways for individuals receiving AI-assisted interpretation in the cost-effectiveness model. 28
Fig. 14s The model selection process. 29
Fig. 15s The comprehensive interpretability analyses. 30
Fig. 16s The cost-effectiveness analysis results presented in terms of the incremental cost-effectiveness ratio per life-year gained.. 32

Supplementary methods

General machine learning process

The machine learning process included the fine-tuning training phase, specific configurations, including batch size, maximum training epochs, loss function, optimizer, dynamic learning rate adjustment, and early stopping; all aimed at ensuring effective convergence. Regarding the hyperparameters, the batch size was set to 32, allowing the model to process 32 images at a time before updating the parameters. The categorical cross-entropy was selected as the loss function. The Adam optimizer was used with an initial learning rate of 0.001. After each training epoch, the model performance was evaluated using the validation dataset, and the validation loss was calculated. The ReduceLROnPlateau function was utilized to monitor the validation loss and dynamically adjust the learning rate. If there was no improvement after 3 consecutive epochs (with a patience of 3), the learning rate was reduced by multiplying it by a factor of 0.2. To avoid unnecessary training time and decrease the risk of overfitting, an early stopping mechanism was implemented. If the validation loss did not decrease after 6 consecutive epochs (with a patience of 6), the training process was stopped, considering that further training would not significantly improve the model performance. The maximum training epochs was set to 50, determining the maximal number of times the model iterated through the entire training dataset. After completing the fine-tuning process, a separate testing dataset was used to evaluate the final performance. The transfer learning was used by fine-tuning pre-trained models to leverage the knowledge and learned features from existing models [1-3].

References

1. Bansal P, Raj G, Choudhury T. Blur image detection using Laplacian operator and Open-CV. Proceedings of the SMART-2016, IEEE Conference ID: 39669.
2. Deng J, Dong W, Socher R, et al. ImageNet: a large-scale hierarchical image database. 2009 *Proceedings of the* IEEE Conference on Computer Vision and Pattern Recognition (CVPR).
3. Kather JN, Weis CA, Bianconi F, et al. Multi-class texture analysis in colorectal cancer histology. Sci Rep 2016;6:27988.

Cost-effectiveness analysis

In this study, AI-assisted interpretation was incorporated into traditional endoscopic examination. The benefits of AI were evaluated based on whether the medical management it triggered improved patient outcomes. These benefits needed to be assessed through long-term follow-up and weighed against the additional costs of medical management to determine their acceptability. A computer-based model was therefore used to simulate the long-term costs, effectiveness, and cost-effectiveness of the new AI intervention. The analysis was conducted from the perspective of a single healthcare payer in Taiwan and involved multiple steps. This evaluation was designed as a complementary component of the AI implementation study. Cost-effectiveness was assessed using empirical data from the present study; however, sensitivity analyses were not performed, which limits the generalizability of the findings. In addition, the assumptions regarding clinical efficacies require validation through longer cohort follow-up or further clinical trials.

<u>Natural course model</u>

The simulation began with each patient undergoing endoscopic examination. The natural course model reflected routine clinical practice without AI-assisted screening and followed the natural history of a hypothetical cohort aged 50–80 years, accounting for mortality from gastric cancer and other causes based on life table data corresponding to life expectancy. The natural history model of gastric cancer was derived from data obtained in a pragmatic randomized clinical trial [1], along with its cost-effectiveness analysis, and has been validated according to the Taiwan mortality registry data [2]. Details of the transition probabilities by age group are presented in

Table 3s. Individuals with *H. pylori* infection were considered to have a sixfold higher risk of developing gastric cancer compared with uninfected individuals [3]. The annual progression rate from premalignant gastric conditions to gastric cancer was set at 0.338% according to a meta-analysis [4].

<u>Decision analysis</u>

The model assumed that individuals began endoscopic examinations at age 50 and simulated a 30-year time horizon, up to age 80, accounting for competing mortality risks based on the life tables. The decision tree Markov models are shown in the **Fig. 12s** (strategies and health status). Two strategies were compared: traditional endoscopic examination and AI-assisted endoscopic examination. In the traditional strategy, routine evaluation for *H. pylori* infection or premalignant gastric conditions was not performed; instead, both were assessed opportunistically using the rapid urease test and histological biopsy. Based on the empirical data, 5% of individuals underwent an opportunistic rapid urease test for *H. pylori*, and premalignant conditions were opportunistically detected in approximately 10% of endoscopies. When either the rapid urease test or histology for premalignant conditions was positive, patients received antibiotic treatment and endoscopic surveillance every three years, respectively.

In the AI-assisted strategy, the system routinely provided information on both *H. pylori* infection and the presence of premalignant gastric conditions (**Fig. 13s**). A positive AI result for *H. pylori* prompted a ^{13}C urea breath test, while a positive result for premalignant conditions led to endoscopic surveillance every three years [5].

<u>Markov cycles</u>

A Markov cycle was a fixed time interval of one year in which patients can either remain in their current health state or transition to another state according to defined probabilities (**Fig. 12s**). At the end of each cycle, the model records outcomes such as costs and life years gained. The process is repeated for multiple cycles, until the entire cohort has reached an absorbing state (death) or for a predetermined time horizon of 30 years.

<u>Data input</u>

Details of the data are provided in **Table 3s**. The baseline prevalence of *H. pylori* infection and premalignant gastric conditions was set at 20% each, based on empirical observations in the study. In the non-AI scenario, 5% of individuals underwent an opportunistic rapid urease test for *H. pylori* (USD 10 per test), followed by eradication therapy. Individuals identified with premalignant gastric conditions (10%) in routine clinical practice received upper endoscopic surveillance every three years, at a cost of USD 50 per examination.

In the AI-assisted scenario, an initial setup cost of USD 1,000 was assigned to cover GPU and other necessary equipment for AI implementation. Based on the parameters generated from this study, the AI model demonstrated a sensitivity and specificity of 95% and 91%, respectively, for detecting *H. pylori*, and 78% and 72%, respectively, for detecting premalignant gastric conditions. Individuals identified as *H. pylori*-positive by AI underwent a ^{13}C urea breath test (USD 35) confirmation and received eradication therapy (USD 42). Those identified with premalignant gastric conditions underwent upper endoscopic surveillance every three years, at a cost of USD 50 per examination.

The study adopted a societal perspective. The cost data included the direct cost and indirect cost. The direct cost data was based on Taiwan Insurance Regulation. Indirect costs were calculated based on the time spent on the screening program and follow-up management, and converted into monetary value using productivity estimates (**Table 3s**).

<u>Assumptions</u>

The model assumed that gastric cancers detected by AI were diagnosed at the earlier stages, resulting in an approximately 50% reduction in the progression-to-death rate compared to the

general rate of gastric cancer [6]. *H. pylori* eradication was associated with about 20% reduction of gastric cancer incidence after adjusting for adherence to invitation [1].

End points
The primary end points were life-years gained, calculated by translating mortality reductions from screening into life-years gained. The incremental cost-effectiveness ratio (ICER) was calculated as the difference in costs divided by the difference in life-years between two strategies. The analyses were performed using TreeAge Pro 2024 (TreeAge Software, Inc., Williamstown, MA, USA).

Supplementary results
The results are shown in the **Fig. 16s.** The benefits of *H. pylori* eradication and early gastric cancer detection triggered by AI may outweigh the burden associated with advanced cancer treatments and reduced life expectancy under the traditional approach. In fact, the AI-assisted approach may achieve lower costs and greater effectiveness in life-years saved, yielding a negative ICER and dominating the traditional approach as a cost-saving strategy.

Nonetheless, the results are based on assumptions about the efficacy of *H. pylori* eradication for gastric cancer prevention and the efficacy of endoscopic surveillance for early detection. The effectiveness of *H. pylori* eradication may be underestimated due to limited adherence to invitations [1], whereas the effectiveness of early cancer detection through endoscopic surveillance may be overestimated given the potentially low uptake of endoscopy [6]. Also, the sensitivity analyses are not carried out. This work should be regarded as an exploratory analysis that aimed at assessing the potential benefits of the AI-assisted approach in a real-life setting. The findings need to be verified through ongoing clinical studies or clinical trials.

Table 1s Recent studies for the use of artificial intelligence to assist in diagnosing *Helicobacter pylori* infection and premalignant conditions using static images or real-time analyses.

A: For the diagnosis of atrophic gastritis.

Study, year	Site	Endoscopy	AI algorithm	Study Design	Patient (n)	Image (n)	Video (n)	Sensitivity (%)	Specificity (%)	Accuracy (%)
Huang, 2004 [1]	Taiwan	WLI	Customized Neural Networks	Retrospective	104	74	-	83.3	82.6	82.9
Zhang, 2020 [2]	China	WLI	DenseNet121	Retrospective	1,699	5,470	-	94.5	94.0	94.2
Guimaraes, 2020 [3]	Germany	WLI	VGG16	Retrospective	35	70	-	100	87.5	92.9
Mu, 2021 [4]	China	WLI	UNet++, ResNet50	Prospective	4,587	8,141	-	-	-	91.2
Xu, 2021 [5]	China	ME-NBI/BLI	VGG16	Prospective	213	1,052	98	89.8	79.4	86.4
Lin, 2021 [6]	China	WLI	TResNet	Retrospective	2,741	7,037	-	96.2	96.4	96.4
Luo, 2022 [7]	China	WLI	ResNet50	Retrospective	4,005	10,593	-	87.0	85.0	85.4
Yang, 2023 [8]	China	WLI, LCI	SE-ResNet50	Retrospective	na	21,420	-	95.3	98.9	97.1
Tao, 2024 [9]	China	WLI	ResNet50	Retrospective	102	869	119	92.7	92.3	92.5

B: For the diagnosis of intestinal metaplasia.

Study, year	Site	Endoscopy	AI algorithm	Study Design	Patient (n)	Image (n)	Video (n)	Sensitivity (%)	Specificity (%)	Accuracy (%)
Huang, 2004 [1]	Taiwan	WLI	Customized Neural Networks	Retrospective	104	74	-	83.3	91.9	90.1
Yan, 2020 [10]	China	NBI, ME-NBI	EfficientNetB4	Retrospective	416	2,357	-	91.9	86.0	88.8
Mu, 2021 [4]	China	WLI	UNet++, ResNet50	Prospective	4,587	8,141	-	na	na	85.8
Xu, 2021 [5]	China	ME-NBI/BLI	VGG16	Prospective	213	1,052	98	96.9	72.3	85.9
Lin, 2021 [6]	China	WLI	TResNet	Retrospective	2,741	7,037	-	97.9	97.5	97.6
Li, 2021 [11]	China	NBI	Improved ResNet	Retrospective	242	1,050	-	93.2	87.1	90.3
Siripoppohn, 2022 [12]	Thailand	WLI, NBI	Improved BiSeNet	Prospective	-	802	32 frames per second	93.1	80.0	86.6
Wong, 2022 [13]	China	ME-NBI	Improved ResNet	Retrospective	420	1,372	-	93.6	91.2	93.2
Yang, 2023 [8]	China	WLI, LCI	SE-ResNet50	Retrospective	-	21,420	-	98.9	99.5	99.2
Pornvoraphat, 2023 [14]	Thailand	WLI, NBI	Improved BiSeNet	Retrospective	-	2,179	173 frames per second	91.4	95.7	94.3

C: For the diagnosis of *H. pylori* infection.

Study, year	Site	Endoscopy	AI algorithm	Study Design	Patient (n)	Image (n)	Video (n)	Sensitivity (%)	Specificity (%)	Accuracy (%)
Huang, 2004 [1]	Taiwan	WLI	Customized Neural Networks	Retrospective	104	74	-	78.8	90.2	85.1
Shichijo, 2017 [15]	Japan	WLI	GoogLeNet	Retrospective	397	11,481	-	88.9	87.4	87.7
Itoh, 2018 [16]	Japan	WLI	GoogLeNet	Prospective	139	179	-	86.7	86.7	86.7
Nakashima, 2018 [17]	Japan	WLI, BLI, LCI	GoogLeNet	Prospective	222	648	-	96.7	86.7	NA
Shichijo, 2019 [18]	Japan	WLI	GoogLeNet	Retrospective	847	23,699	-	62.9	94.0	91.4
Zheng, 2019 [19]	China	WLI	ResNet50	Retrospective	452	3755	-	91.6	98.6	93.8
Nakashima, 2020 [20]	Japan	WLI, BLI, LCI	CAD system	Prospective	395	12,887	120	61.3	89.4	80.0
Yasuda, 2020 [21]	Japan	LCI	SVM	Retrospective	105	525	-	90.5	85.7	87.6
Yoshii, 2020 [22]	Japan	WLI	NM	Prospective	498	-	-	67.1	91.4	82.9
Zhang, 2023 [23]	China	WLI	ResNet50	Retrospective	1,826	47,239	-	92.9	89.3	91.1
Lin, 2023 [24]	Taiwan	WLI	CNN, scSE networks	Retrospective	302	959	-	100	81	90
Seo, 2023 [25]	Korea	WLI	CNN	Retrospective	952	13,403	-	66	87	71
Li, 2023 [26]	China	WLI	CNN	Retrospective	191	NA	100	82	86	84
Zou, 2024 [27]	China	WLI	CNN	Prospective	639	7,377	-	90.9	88.9	89.9

Abbreviation: AI = Artificial Intelligence; WLI = White Light Imaging; ME= Magnifying Endoscopy; NBI = Narrow-Band Imaging; BLI = Blue Laser Imaging; LCI = Linked Color Imaging

References:

1. Huang CR, Sheu BS, Chung PC et al. Computerized diagnosis of *Helicobacter pylori* infection and associated gastric inflammation from endoscopic images by refined feature selection using a neural network. Endoscopy 2004; 36: 601-608.
2. Zhang Y, Li F, Yuan F, Zhang K et al. Diagnosing chronic atrophic gastritis by gastroscopy using artificial intelligence. Dig Liver Dis 2020; 52: 566-572.
3. Guimaraes P, Keller A, Fehlmann T et al. Deep-learning based detection of gastric precancerous conditions. Gut. 2020;69:4-6.
4. Mu G, Zhu Y, Niu Z, et al. Expert-level classification of gastritis by endoscopy using deep learning: a multicenter diagnostic trial. Endosc Int Open 2021;9:E955-E964.
5. Xu M, Zhou W, Wu L et al. Artificial intelligence in the diagnosis of gastric precancerous conditions by image-enhanced endoscopy: a multicenter, diagnostic study (with video). Gastrointest Endosc 2021; 94: 540-548 e4.
6. Lin N, Yu T, Zheng W et al. Simultaneous recognition of atrophic gastritis and intestinal metaplasia on white light endoscopic images based on convolutional neural networks: A multicenter study. Clin Transl Gastroenterol 2021; 12: e00385.
7. Luo J, Cao S, Ding N et al. A deep learning method to assist with chronic atrophic gastritis diagnosis using white light images. Dig Liver Dis 2022; 54: 1513-9.
8. Yang J, Ou Y, Chen Z et al. A benchmark dataset of endoscopic images and novel deep learning method to detect intestinal metaplasia and gastritis atrophy. IEEE J Biomed Health Inform 2023; 27: 7-16.
9. Tao X, Zhu Y, Dong Z, Huang L et al. An artificial intelligence system for chronic atrophic gastritis diagnosis and risk stratification under white light endoscopy. Dig

Liver Dis 2024; 56: 1319-26.
10. Yan T, Wong PK, Choi IC et al. Intelligent diagnosis of gastric intestinal metaplasia based on convolutional neural network and limited number of endoscopic images. Comput Biol Med 2020; 126: 104026.
11. Li HY, Vong CM, Wong PK et al. A multi-feature fusion method for image recognition of gastrointestinal metaplasia (GIM). Biomedical Signal Processing and Control 2021; 69.
12. Siripoppohn V, Pittayanon R, Tiankanon K et al. Real-time semantic segmentation of gastric intestinal metaplasia using a deep learning approach. Clin Endosc 2022; 55: 390-400.
13. Wong PK, Yao L, Yan T et al. Broad learning system stacking with multi-scale attention for the diagnosis of gastric intestinal metaplasia. Biomedical Signal Processing and Control 2022; 73.
14. Pornvoraphat P, Tiankanon K, Pittayanon R et al. Real-time gastric intestinal metaplasia diagnosis tailored for bias and noisy-labeled data with multiple endoscopic imaging. Comput Biol Med 2023; 154: 106582.
15. Shichijo S, Nomura S, Aoyama K et al. Application of convolutional neural networks in the diagnosis of *Helicobacter pylori* infection based on endoscopic images. EBioMedicine 2017; 25: 106-111.
16. Itoh T, Kawahira H, Nakashima H et al. Deep learning analyzes *Helicobacter pylori* infection by upper gastrointestinal endoscopy images. Endosc Int Open 2018; 6: E139-E144.
17. Nakashima H, Kawahira H, Kawachi H et al. Artificial intelligence diagnosis of *Helicobacter pylori* infection using blue laser imaging-bright and linked color imaging: a single-center prospective study. Ann Gastroenterol 2018;31:462-468.
18. Shichijo S, Endo Y, Aoyama K et al. Application of convolutional neural networks for evaluating *Helicobacter pylori* infection status on the basis of endoscopic images. Scand J Gastroenterol 2019; 54: 158-163.
19. Zheng W, Zhang X, Kim JJ et al. High accuracy of convolutional neural network for evaluation of *Helicobacter pylori* infection based on endoscopic images: Preliminary experience. Clin Transl Gastroenterol 2019; 10: e00109.
20. Nakashima H, Kawahira H, Kawachi H et al. Endoscopic three-categorical diagnosis of *Helicobacter pylori* infection using linked color imaging and deep learning: a single-center prospective study (with video). Gastric Cancer 2020; 23: 1033-1040.
21. Yasuda T, Hiroyasu T, Hiwa S et al. Potential of automatic diagnosis system with linked color imaging for diagnosis of *Helicobacter pylori* infection. Dig Endosc 2020; 32: 373-381.
22. Yoshii S, Mabe K, Watano K, et al. Validity of endoscopic features for the diagnosis of *Helicobacter pylori* infection status based on the Kyoto classification of gastritis. Dig Endosc 2020; 32: 74-83.
23. Zhang M, Pan J, Lin J et al. An explainable artificial intelligence system for diagnosing *Helicobacter pylori infe*ction under endoscopy: a case-control study. Therap Adv Gastroenterol 2023;16:17562848231155023.
24. Lin CH, Hsu PI, Tseng CD et al. Application of artificial intelligence in endoscopic image analysis for the diagnosis of a gastric cancer pathogen-*Helicobacter pylori* infection. Sci Rep 2023; 13: 13380.
25. Seo JY, Hong H, Ryu WS et al. Development and validation of a convolutional neural network model for diagnosing *Helicobacter pylori* infections with endoscopic images: a multicenter study. Gastrointest Endosc 2023; 97: 880-888 e2.
26. Li YD, Wang HG, Chen SS et al. Assessment of *Helicobacter pylori* infection by deep learning based on endoscopic videos in real time. Dig Liver Dis 2023; 55: 649-654.
27. Zou PY, Zhu JR, Zhao Z et al. Development and application of an artificial intelligence-assisted endoscopy system for diagnosis of *Helicobacter pylori* infection: a multicenter randomized controlled study. BMC Gastroenterol 2024; 24: 335.

Table 2s Baseline characteristics of patients with intragastric histological results for model development.

**Baseline characteristics**	**Medical center (*n*=1175)**	**Rural hospital (*n*=561)**
Age, years (standard deviation; range)	52.0 (13.3; 21-84)	53.7 (12.2; 30-88)
Male sex, no. (%)	449 (38.2)	283 (53.3)
Intra-gastric histopathology, no. (%)		
Atrophic gastritis of antrum	926 (78.8)	215 (38.3)
Atrophic gastritis of corpus	160 (13.6)	105 (18.7)
OLGA		
Stage 0	232 (19.7)	276 (49.2)
Stage 1	472 (40.2)	229 (40.8)
Stage 2	356 (30.3)	22 (3.9)
Stage 3	106 (9.0)	4 (0.7)
Stage 4	9 (0.8)	0
Intestinal metaplasia of antrum	218 (18.6)	174 (31.0)
Intestinal metaplasia of corpus	40 (3.4)	33 (5.9)
OLGIM		
Stage 0	943 (80.2)	339 (60.4)
Stage 1	143 (12.2)	102 (18.2)
Stage 2	73 (6.2)	63 (11.2)
Stage 3	14 (1.2)	20 (3.6)
Stage 4	2 (0.2)	7 (1.2)

Abbreviation: OLGA = Operative Link for Atrophic Gastritis Assessment; OLGIM = Operative Link for Intestinal Metaplasia Assessment; Medical center = National Taiwan University Hospital, a medical center; rural hospital = Lienchiang County Hospital on Matsu Islands, a district hospital on an offshore island

The histological data of 60 patients were not used for model development due to the poor endoscopic image quality.

Table 3s Data for the cost-effectiveness analysis.

Variable	Data	Source
Natural history and prognosis per year, %		
Transition probabilities of gastric carcinogenesis		
Age specific gastric cancer incidence rates		[7]
at 50–54 years	0.01437	
at 55–59 years	0.02334	
at 60–64 years	0.03268	
at 65–69 years	0.04608	
at 70–74 years	0.06068	
at 75–79 years	0.08531	
Relative risk of GC for *H. pylori* infection	6	[3]
Reinfection rate of *H. pylori* after successful eradication	0.30	[1]
Progression rate from premalignant conditions to GC	0.338	[4]
GC to death	0.16	[8]
Other causes of death	Life table	[9]
Prevalence rate at 50 years of age, %		
H. pylori infection	20	[10]
Premalignant gastric conditions	20	[10]
Screening and treatment, %		
Traditional upper endoscopic examination		
Opportunistic rapid urease test	5	[11]
Opportunistic histological assessment	10	[11]
Routine AI-assisted interpretation		
Detecting *H. pylori* infection		[10]
Sensitivity	95	
Specificity	91	
Routine AI-assisted interpretation		
Detecting gastric premalignant conditions		[10]
Sensitivity	78	
Specificity	72	
Eradication rate for *H. pylori*	92	[1]
Efficacies, %		
Reduction of GC after successful *H. pylori* eradication	20	[1]
Reduced progression to GC related death via surveillance endoscopy from early detection	50	[6]
Direct costs, US $		
^{13}C urea breath test	35	[12]
Antibiotic treatment for *H. pylori* eradication	42	[12]
Upper endoscopy	50	[12]
Rapid urease test	10	[12]
AI implementation, including GPU	1,000	[10]
Initial management of GC	9,750	[13]
Continuing treatment for advanced GC	294	[13]
Terminal GC care	8,938	[13]
Indirect costs, US $		
Screening time, hour	0.5	[1]
Person accompanied for screening	0	[1]
Confirmation time, hour	1	[1]
Person accompanied for confirmation	0.2	[1]
Inpatient hospitalization, day	21	[13]
Inpatient recovery at home, day	20	[13]
Person accompanied for inpatient care	1.25	[13]
Outpatient time per visit, hour	4	[13]
Outpatient visits per year	4.68	[13]
Person accompanied for outpatient visit	0.2	[13]
Average monthly work, hour	182	[14]
Production value per hour, US $	15.3	[14]
Average GNP per person, US $	33,365	[14]
Discount rate, %	3	[15]

Abbreviation: GC = Gastric Cancer; GPU = Graphics Processing Unit; GNP = Gross National Product

References:

1. Lee YC, Chiang TH, Chiu HM, et al; Collaborators of Taiwan Community-based Integrated Screening Group. Screening for Helicobacter pylori to prevent gastric cancer: A pragmatic randomized clinical trial. JAMA 2024; 332: 1642-1651.
2. Lee YC, Liu JH, Chiu HM, et al. Cost-effectiveness of fecal immunochemical test and Helicobacter pylori stool antigen co-testing. Gastroenterology 2025; 169: Supplement S-43.
3. de Martel C, Ferlay J, Franceschi S, et al. Global burden of cancers attributable to infections in 2008: a review and synthetic analysis. Lancet Oncol 2012; 13: 607-615.
4. Akbari M, Tabrizi R, Kardeh S, et al. Gastric cancer in patients with gastric atrophy and intestinal metaplasia: A systematic review and meta-analysis. PLoS One 2019; 14: e0219865.
5. Dinis-Ribeiro M, Libânio D, Uchima H, et al. Management of epithelial precancerous conditions and early neoplasia of the stomach (MAPS III): European Society of Gastrointestinal Endoscopy (ESGE), European Helicobacter and Microbiota Study Group (EHMSG) and European Society of Pathology (ESP) Guideline update 2025. Endoscopy 2025; 57: 504-554.
6. Xia C, Li H, Xu Y, et al. Effect of an endoscopy screening on upper gastrointestinal cancer mortality: A community-based multicenter cluster randomized clinical trial. Gastroenterology. 2025; 168: 725-740.
7. Taiwan Cancer Registration Data Explore, Health Promotion Administration, Ministry of Health and Welfare, Taiwan. Available at: https://cris.hpa.gov.tw/EngPages.
8. Cause of Death Statistics, Ministry of Health and Welfare, Republic of China (Taiwan). Available at: https://www.mohw.gov.tw/np-128-2.html.
9. Life tables, Ministry of Interior, Republic of China (Taiwan). Available at: https://www.moi.gov.tw/english/cl.aspx?n=7779.
10. Chiang TH, Hsu YN, Chen MH, et al. A rural-to-center artificial intelligence model for diagnosing Helicobacter pylori infection and premalignant gastric conditions using endoscopy images captured in routine practice. Endoscopy 2025 (the present study).
11. Chiang TH, Chang WJ, Chen SL, et al. Mass eradication of Helicobacter pylori to reduce gastric cancer incidence and mortality: a long-term cohort study on Matsu Islands. Gut 2021; 70: 243-250.
12. National Health Insurance Administration, Ministry of Health and Welfare, Taiwan. Available at: https://www.nhi.gov.tw/en/mp-2.html.
13. Lee YC, Chao YT, Lin PJ, et al. Quality assurance of integrative big data for medical research within a multihospital system. J Formos Med Assoc 2022; 121: 1728-1738.
14. Directorate General of Budget, Accounting and Statistics, Executive Yuan, R.O.C. (Taiwan). Available at: https://eng.dgbas.gov.tw/.
15. Discount rate. Central Bank of Republic of China (Taiwan). Available at: https://www.cbc.gov.tw/en/lp-695-2.html.

Table 4s Comprehensive interpretation analyses in gastric cancer risk evaluation, including the age, sex, and AI assisted diagnoses of active *H. pylori* infection and premalignant conditions, based on the logistic regression model.

Variables included in the risk assessment models	Sensitivity (95% CI)	Specificity (95% CI)	Accuracy (95% CI)
Age and sex	75.5% (67.5%–83.5%)	59.4% (50.9%–68.0%)	73.0% (64.8%–81.2%)
Age, sex, and active *H. pylori* infection	81.6% (74.3%–89.0%)	66.7% (58.2%–75.1%)	82.6% (75.3%–89.8%)
Age, sex, active *H. pylori* infection, AG (corpus and antrum), and IM (corpus and antrum)	89.8% (83.9%–95.7%)	81.6% (74.3%–88.9%)	90.2% (84.4%–96.0%)

Abbreviation: CI = Confidence Interval; AG = Atrophic Gastritis; IM = Intestinal Metaplasia

Table 5s Baseline characteristics of the participants for the screening programs in the model implementation phase.

**Baseline data**	**2023 (*n*=2,651)**	**2024 (*n*=2,855)**
Mean age, years (SD; range)	54.0 (13.4; 30-95)	54.4 (13.6; 30-95)
Male sex, no. (%)	1,267 (47.8)	1312 (45.6)
Body mass index, kg/m^2 (SD; range)	25.3 (4.2; 16.0-52.1)	25.6 (4.2; 16.6-51.6)
Social habits, no. (%)		
Current smoker	406 (15.3)	399 (14.0)
Regular alcohol drinking	167 (6.3)	164 (5.7)
Betel nut chewing	78 (2.9)	75 (2.6)
Medical history, no. (%)		
Hypertension	774 (29.2)	844 (29.6)
Diabetes mellitus	254 (9.6)	284 (9.9)
Hyperlipidemia	268 (10.1)	306 (10.7)
Cardiovascular disease or stroke	115 (4.3)	157 (5.5)
Chronic hepatitis B or C	358 (13.5)	397 (13.9)
Chronic kidney disease	15 (0.6)	23 (0.8)
Personal cancer history, no. (%)		
Lung cancer	2 (0.1)	3 (0.1)
Liver cancer	2 (0.1)	2 (0.1)
Esophageal cancer	0	0
Gastric cancer	3 (0.1)	3 (0.1)
Colorectal cancer	0	0
Family cancer history, no. (%)		
Lung cancer	6 (0.2)	7 (0.2)
Liver cancer	58 (2.2)	80 (2.8)
Esophageal cancer	14 (0.5)	18 (0.6)
Gastric cancer	91 (3.4)	108 (3.8)
Colorectal cancer	44 (1.7)	52 (1.8)

Abbreviation: SD = Standard Deviation

Fig. 1s The geographical distance between the Matsu Islands and Taiwan.
The Matsu Islands are an archipelago of five major islands in the East China Sea: Nangan, Beigan, Dongyin, Western Juguang, and Eastern Juguang. They are located approximately 206 kilometers off the coast of Taiwan, across the Taiwan Strait, near the northern coast of Fujian Province, China. Before 2004, the incidence rate of gastric cancer in the Matsu Islands was approximately 50 per 100,000 person-years, which was 3–5 times higher than the incidence rate on the main island of Taiwan. This alarming disparity led to the implementation of a series of gastric cancer prevention programs. Source: IARC *Helicobacter pylori* Working Group (2014). *Helicobacter pylori* Eradication as a Strategy for Gastric Cancer Prevention. Lyon, France: International Agency for Research on Cancer (IARC Working Group Reports, No. 8).

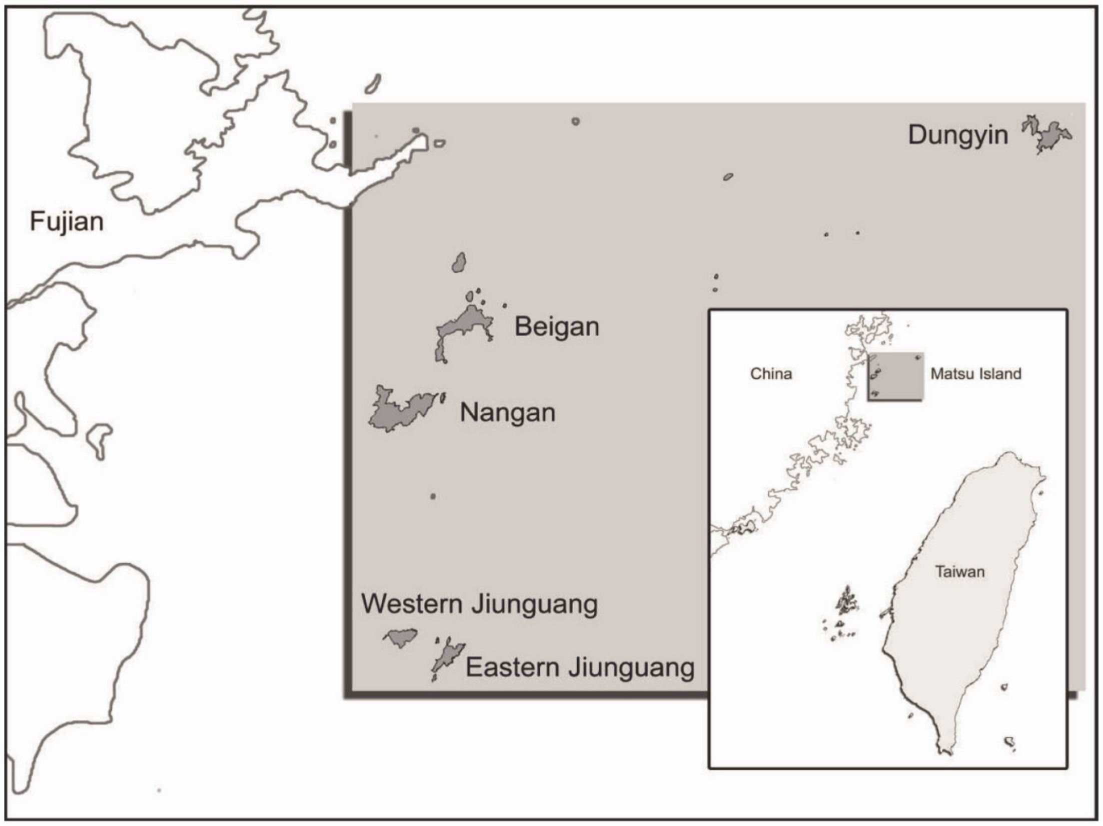

Fig. 2s Preprocessing of upper endoscopic images.
Before proceeding to the deep learning stage, image preprocessing was necessary to address certain issues present in the original images, such as patient information, procedure timing, and watermarks, which were irrelevant to the clinical judgement. Additionally, the images were of varying sizes, including dimensions of 720×480, 640×480, or 512×384 pixels, obtained from GIF Q260, GIF H260, or GIF H290 upper endoscopies (Olympus Corporation, Tokyo, Japan) during the study period. To eliminate these irrelevant artifacts and focus solely on the mucosal appearance, which contained the essential information, the following preprocessing steps were performed on the original image:

(1) Convert RGB color endoscopic images to grayscale images;
(2) Utilize Otsu thresholding [1] to determine the optimal threshold by considering the minimum variance within the same class and the maximum variance between different classes, then convert the grayscale image into a binary image;
(3) Automatically detect the edges of objects in the image and identify the region with the largest area (determined by the maximum and minimum coordinates in the x and y axes;
(4) Extract the desired rectangular region for analysis;
(5) Resize the extracted image to achieve square pixel dimensions (256×256 or 128×128). These preprocessing steps ensured that the images were appropriately prepared for utilization in the subsequent deep learning stage.

Reference:

1. Otsu N. A threshold selection method from gray-level histograms. IEEE Transactions on Systems, Man, and Cybernetics 1979; 9: 62-66.

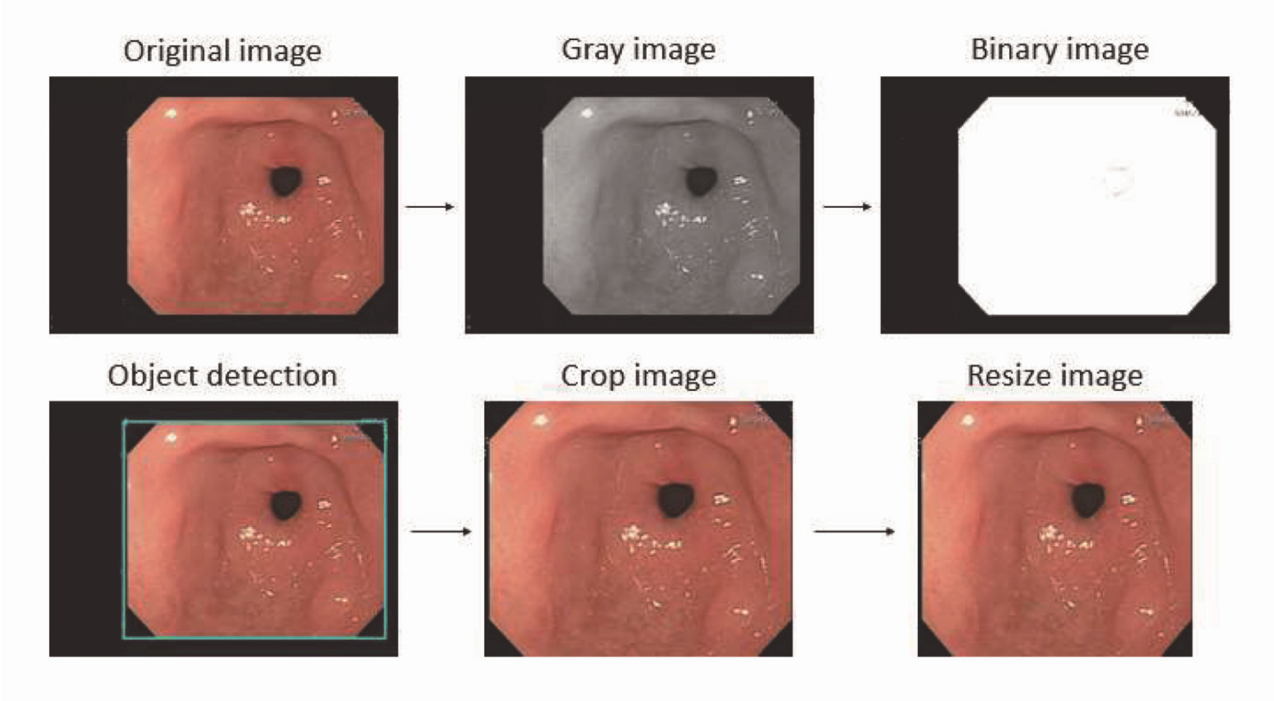

Fig. 3s Exclusion of the blurred images.
The Laplacian method was utilized to detect blurred images by analyzing the image variance, which highlights edge details. Clear images show distinct edges, resulting in higher variance, while blurry images lack edge information, leading to lower variance. A total of 1036 blurred images and 865 clear images were selected by the experienced endoscopists. A threshold of 800 was set based on clinical judgement. Images with Laplacian scores below 800 are considered blurred images and will be excluded, while those scoring 800 or above are considered clear and will be used in the model. The distribution of Laplacian scores for manually selected blurred versus clear images are shown in A. Illustration of blurred and clear images selection is shown in B.

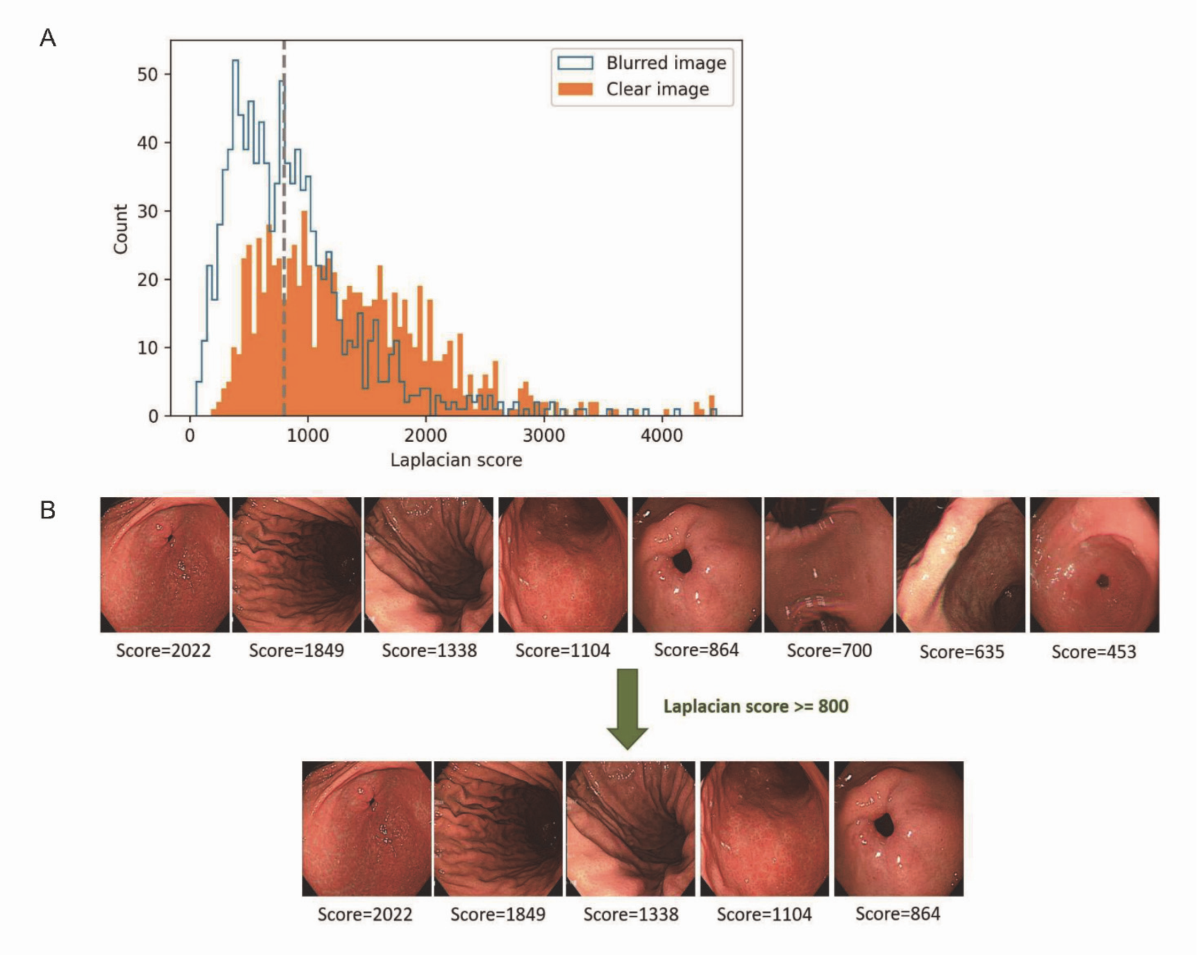

Endoscopy | DOI 10.1055/a-2721-6552 | © The Author(s).

Fig. 4s Rationale for the artificial intelligence models using the histological prediction model as an example.
The system automatically uploads raw and stored endoscopic images, processes them by removing non-stomach images, delineates stomach areas, enhances the images, predicts histological outcomes, and displays the results with heatmaps. The heatmaps use a color spectrum where red indicates regions that received more attention from the model, while blue indicates regions that received less attention.

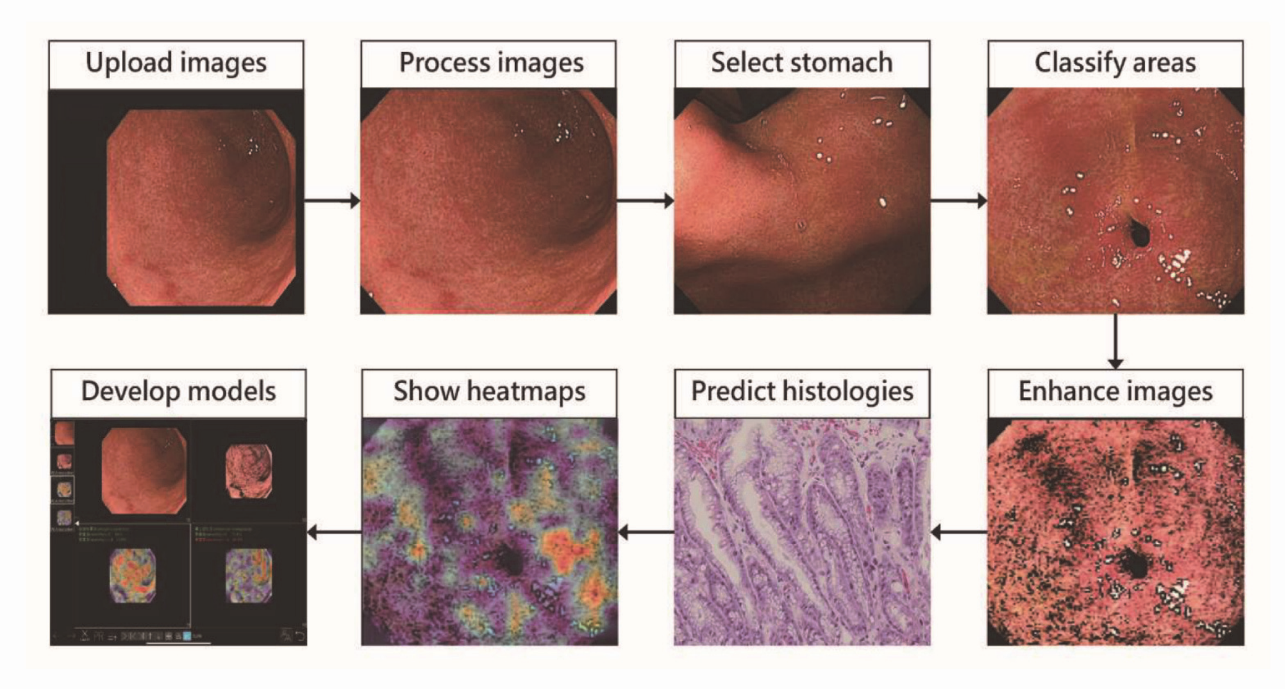

Fig. 5s Exclusion of enhanced images and organic lesions.
The categories of normal images (A), enhanced images (B), and organic lesions, including polyps (C), tumors (D), and ulcers (E), were manually categorized, as shown in the figures. Grad-CAM took the weights from the last convolutional layer using backpropagation to calculate the CAM. It presented a heatmap (16 ×16), where features to which the model paid more attention were represented with colors closer to yellow, while areas with which the model were less concerned were shown in darker blue. Subsequently, the heatmap colors were modified using the commonly-used mapping algorithm COLORMAP_JET. In this modification, features to which the model was more attentive were depicted in shades closer to red, while areas with less model attention were displayed in shades closer to blue. The heatmap is then resized to 256 × 256 or 128 × 128 and overlaid onto the endoscopic image.

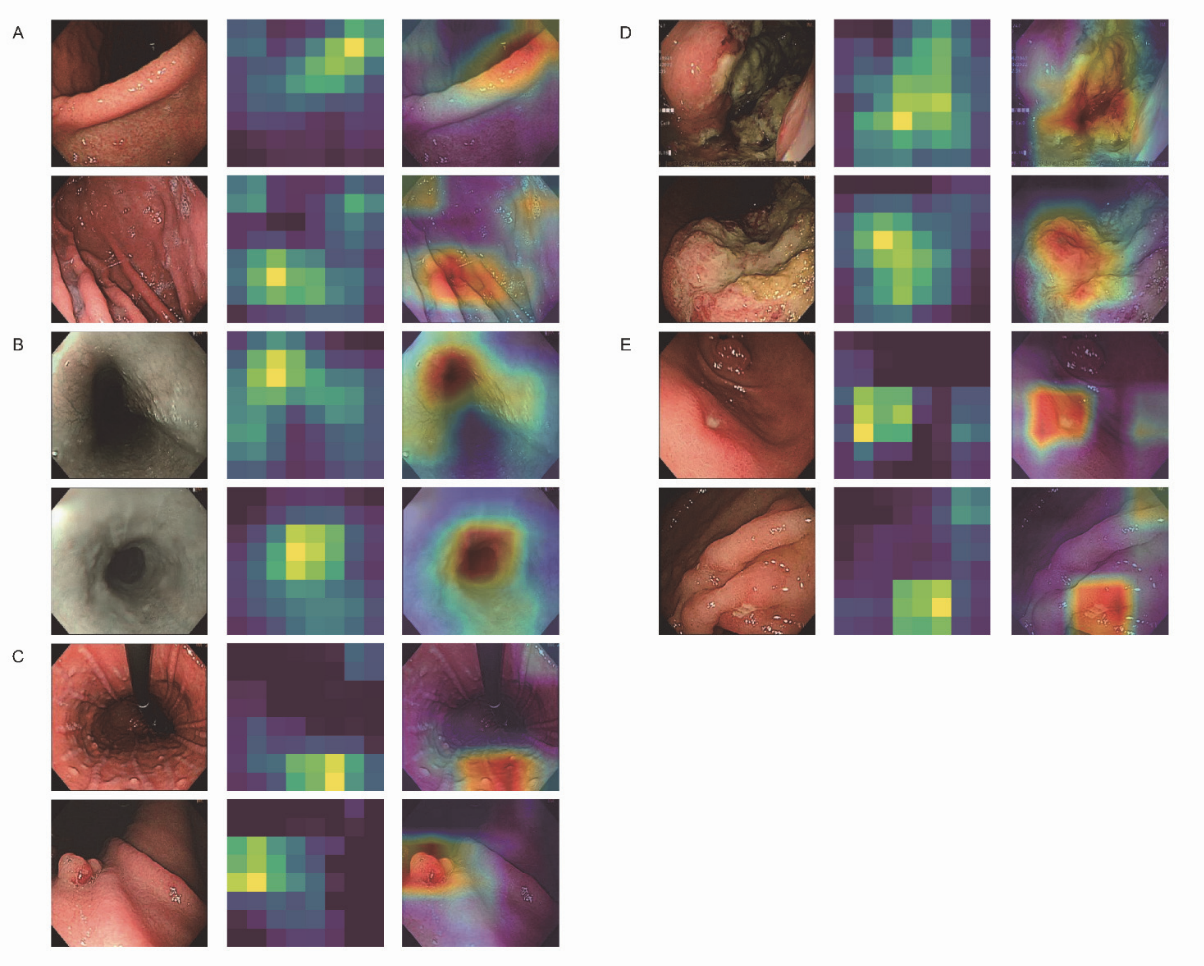

Fig. 6s Classification of upper endoscopy images according to the non-stomach images and stomach images.

For stomach image recognition, the Grad-CAM was also used to visualize whether the model focuses on the correct regions. Grad-CAM highlighted areas of an image critical to the model's decision-making process, helping validate if the model directs attention to relevant regions for accurate classification. The visualization results showed predicted areas divided into non-gastric (hypopharynx, esophagus, duodenum) (A) and gastric regions (B), with each region displaying two results. The left image shows the endoscopic view, and the right shows the Grad-CAM overlay. The heatmap uses COLORMAP_JET, with red indicating high attention and blue indicating low attention. For example, for non-gastric areas, the model focused on regions like the vocal folds, esophagus, or duodenum, depending on the image type. For gastric areas, the model may focus on the pyloric ring.

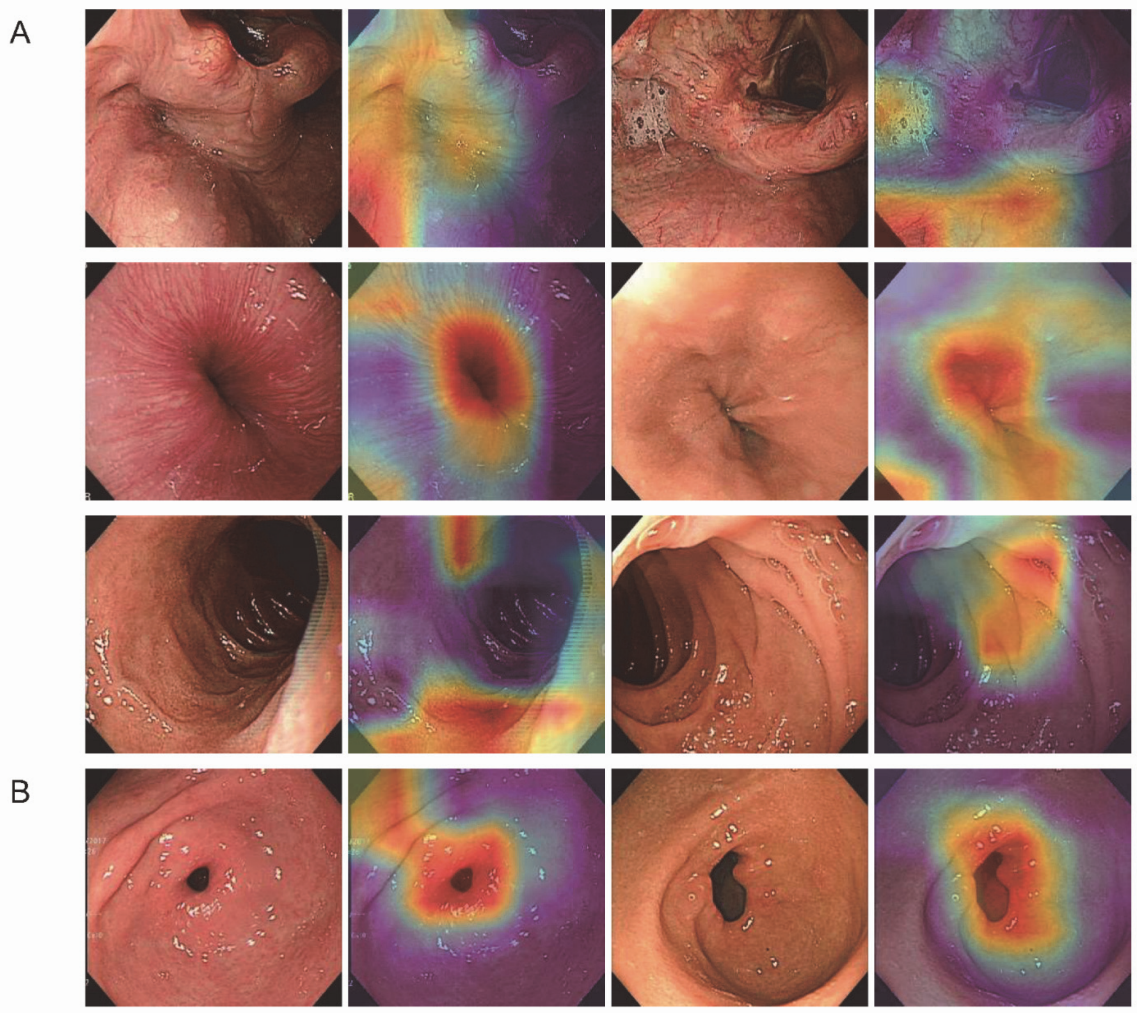

Fig. 7s Classification of stomach images according to the antrum, corpus, and cardia/fundus. For gastric anatomical recognition, Grad-CAM was also used to visualize whether the models focus on the relevant areas. The process was similar to non-gastric region recognition. When the ground truth indicated a gastric location and the image showed the gastric antrum, the model targeted the pylorus, folds, or areas with varying depth (A). For the gastric corpus, the model focuses on the mucosal folds (B). For the cardia/fundus, the model highlights recessed regions or the junction of esophagogastric structure (C).

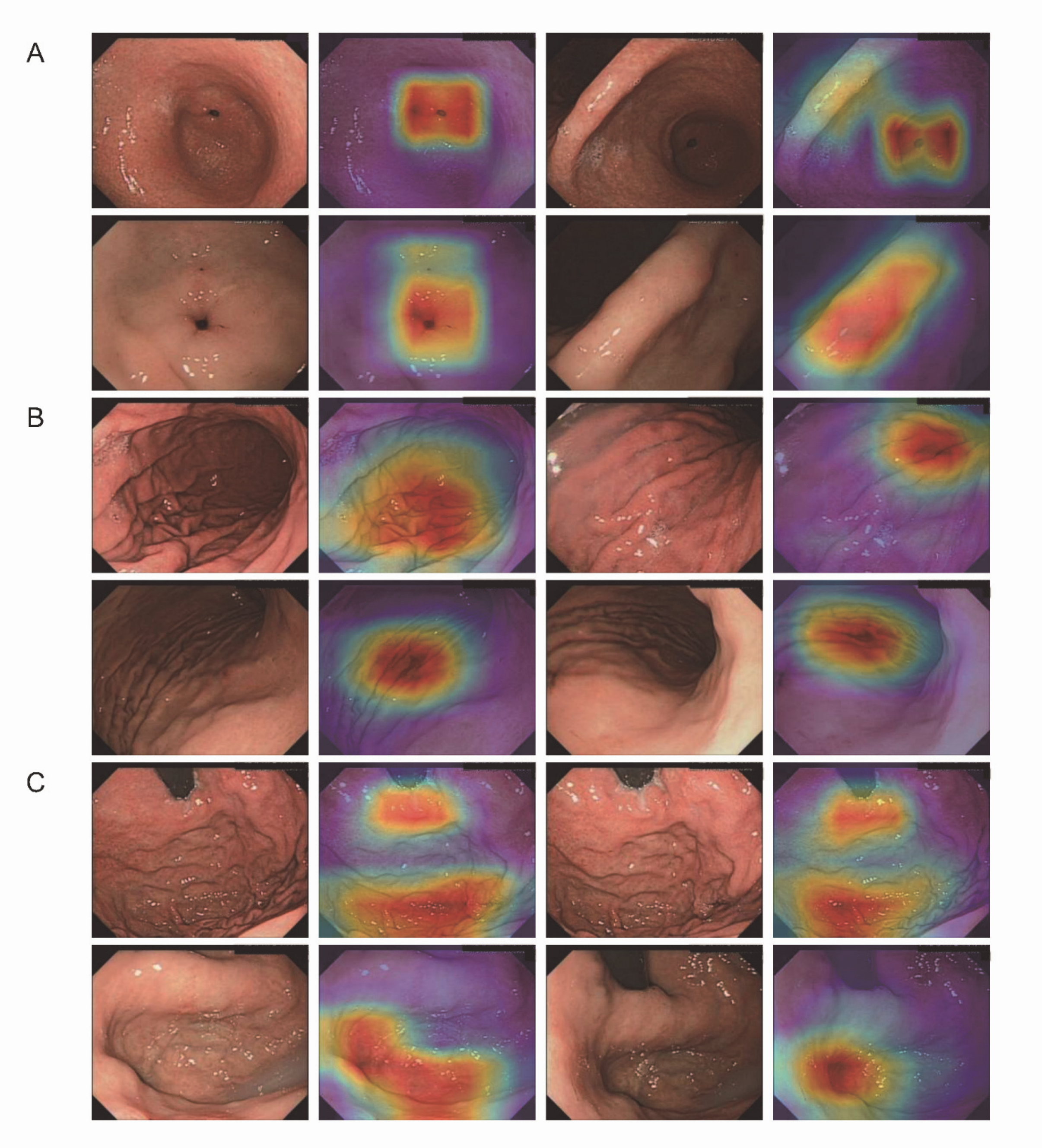

Fig. 8s Mucosal patterns of *H. pylori* infection according to the anatomical locations. Gastric mucosal patterns associated with *H. pylori* infection include diffuse redness, mucosal swelling, and nodularity. The anatomical locations of the illustrated images are the fundus/cardia (A), corpus (B), and antrum (C).

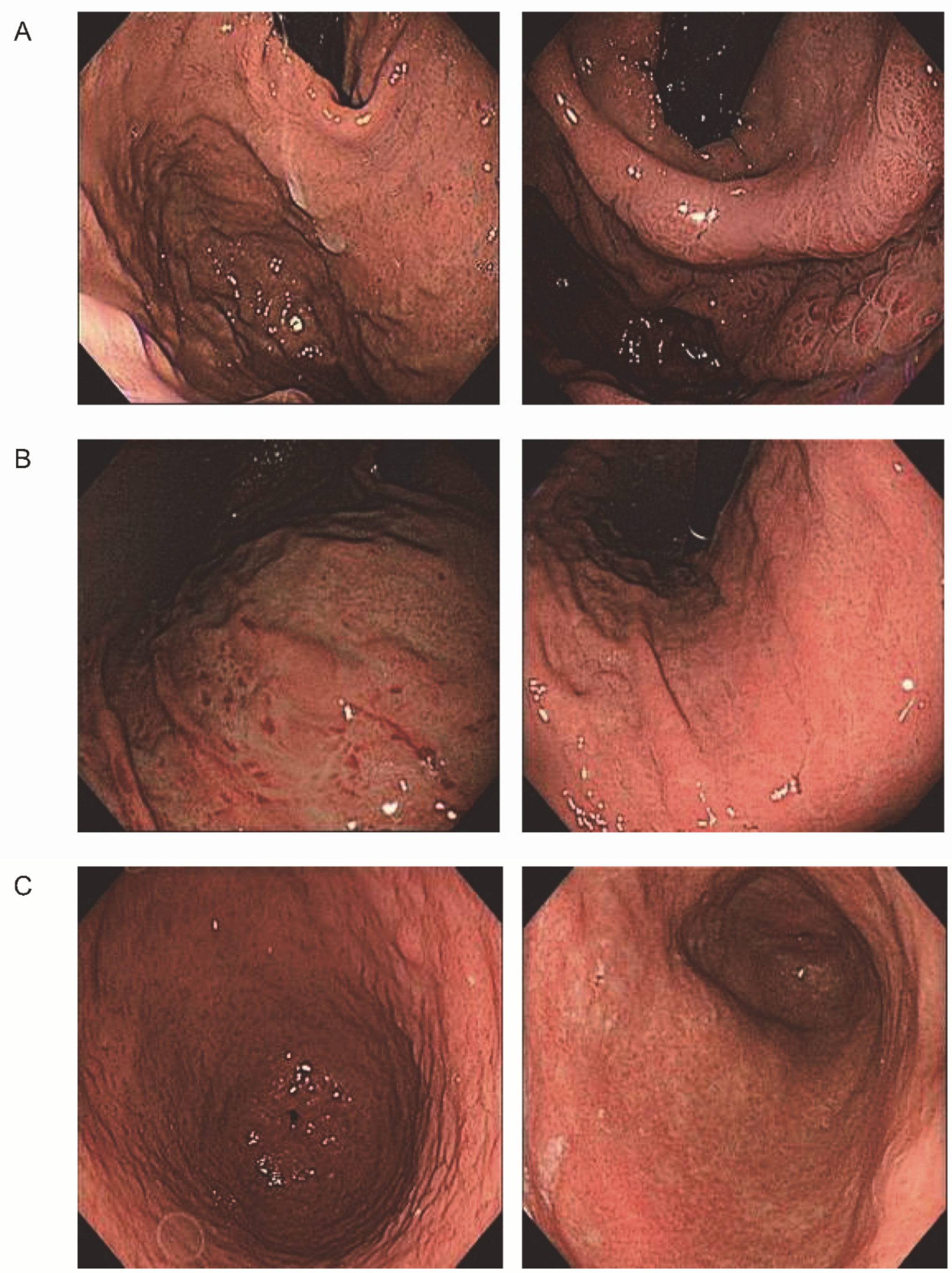

Fig. 9s Classification of stomach images according to the presence of *H. pylori* infection.
In this stage, model training using the original white-light images, CLAHE-enhanced images, and dynamic threshold white-balanced images were evaluated. The white-balanced images performed better. Dynamic threshold white balancing involved converting the RGB space to the YUV space, where Y represented brightness and UV represents chrominance. Adjustments were made using dynamic thresholds for white point detection, with white as the base color. Models trained with dynamically threshold white-balance images demonstrated superior performance compared to those trained with original and CLAHE-enhanced images (A). This method enhanced vascular patterns and structural changes in the gastric mucosa, aiding the model in predicting mucosal inflammation.

The classification process (B: *H. pylori*-negative images and C: active *H. pylori* infection images) began with image enhancement using white balance, followed by deep learning techniques, and finally, the generation of heatmaps using Grad-CAM. Grad-CAM took the weights from the last convolutional layer using backpropagation to calculate the CAM. Similarly, it presented a heatmap (16 ×16), where features to which the model pays more attention are represented with colors closer to yellow, while areas with which the model is less concerned were shown in darker blue as mentioned previously.

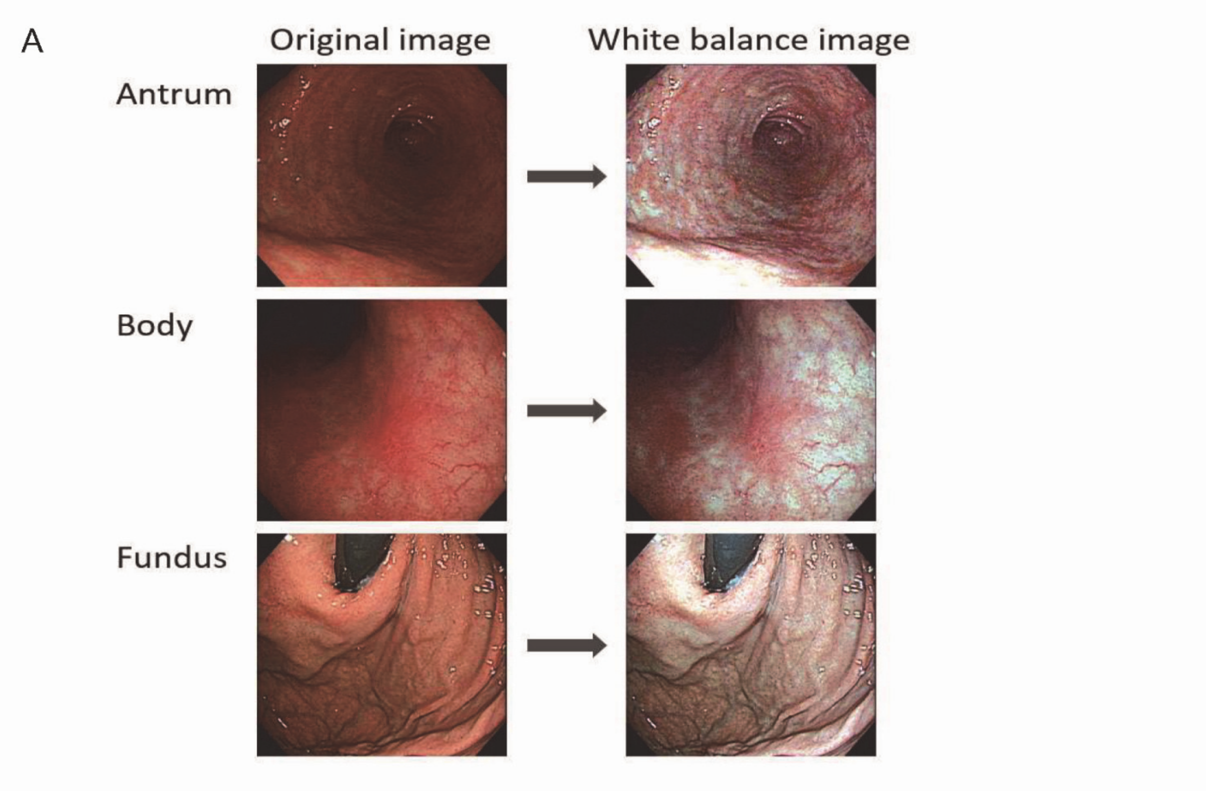

Figure 9b and c

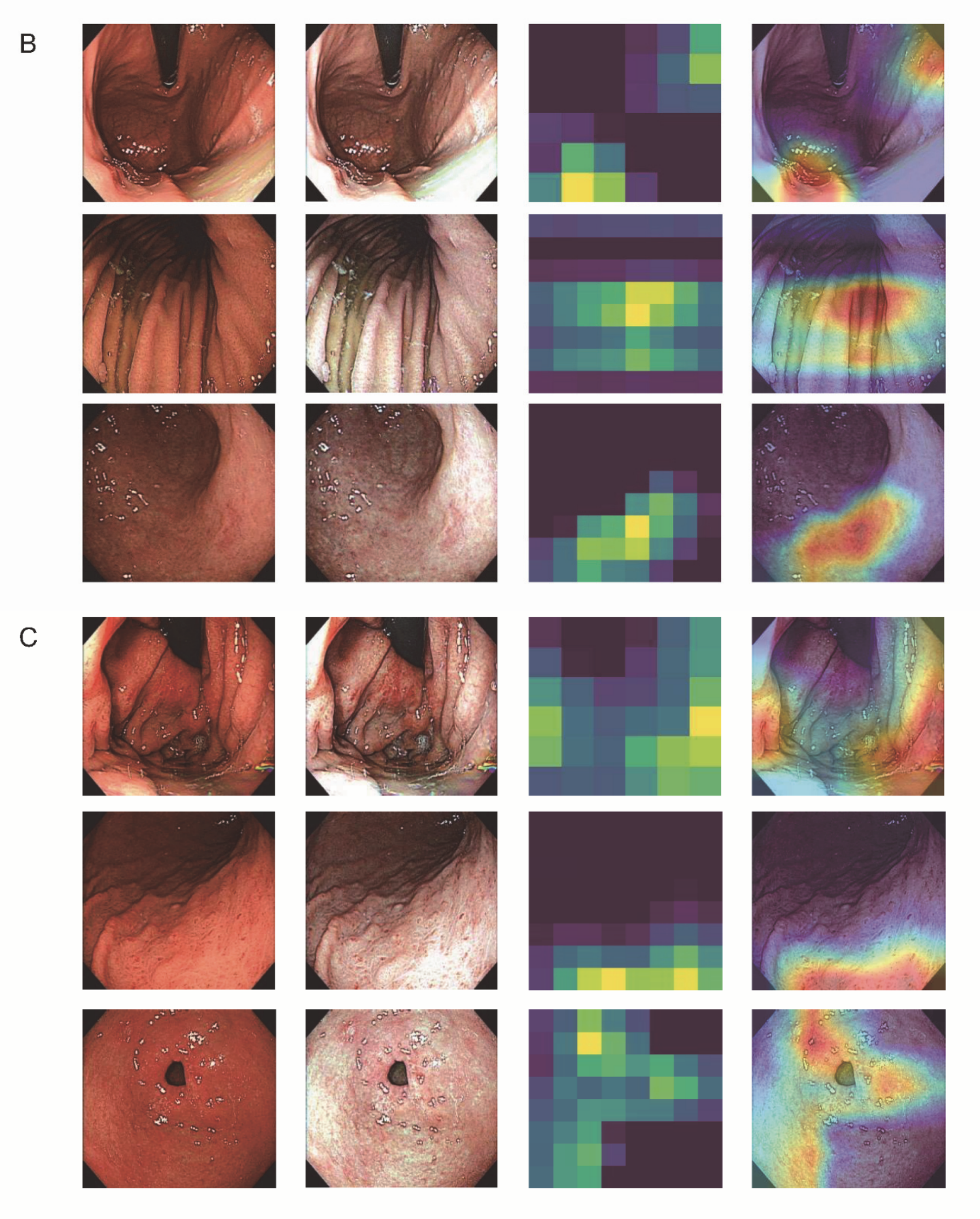

Endoscopy | DOI 10.1055/a-2721-6552 | © The Author(s).

Fig. 10s Classification of stomach images according to the presence of premalignant gastric conditions.
At this stage, image enhancement was needed to highlight the premalignant condition. Contrast limited adaptive histogram equalization (CLAHE) was applied, as illustrated in following figure. CLAHE involved constraining the contrast within small regions to enhance the features of the nearby gastric mucosa, obtaining more information (A). The resulting images closely emulated the characteristics of clinical diagnostic images, similar to the process of image-enhanced endoscopy in practice, facilitating the evaluation of mucosal patterns through computer virtual contrast enhancement.

For premalignant gastric condition recognition (B: atrophic gastritis and C: intestinal metaplasia), the classification process began with image enhancement using CLAHE, followed by deep learning techniques, and finally, the generation of heatmaps using Grad-CAM visualizations. Grad-CAM took the weights from the last convolutional layer using backpropagation to calculate the CAM. It presented a heatmap (16 ×16), where features to which the model paid more attention were represented with colors closer to yellow, while areas with which the model is less concerned are shown in darker blue. Subsequently, the heatmap colors were modified using the commonly-used mapping algorithm COLORMAP_JET. In this modification, features to which the model was more attentive were depicted in shades closer to red, while areas with less model attention were displayed in shades closer to blue. The heatmap was then resized to 256 × 256 or 128 × 128 and overlaid onto the endoscopic image.

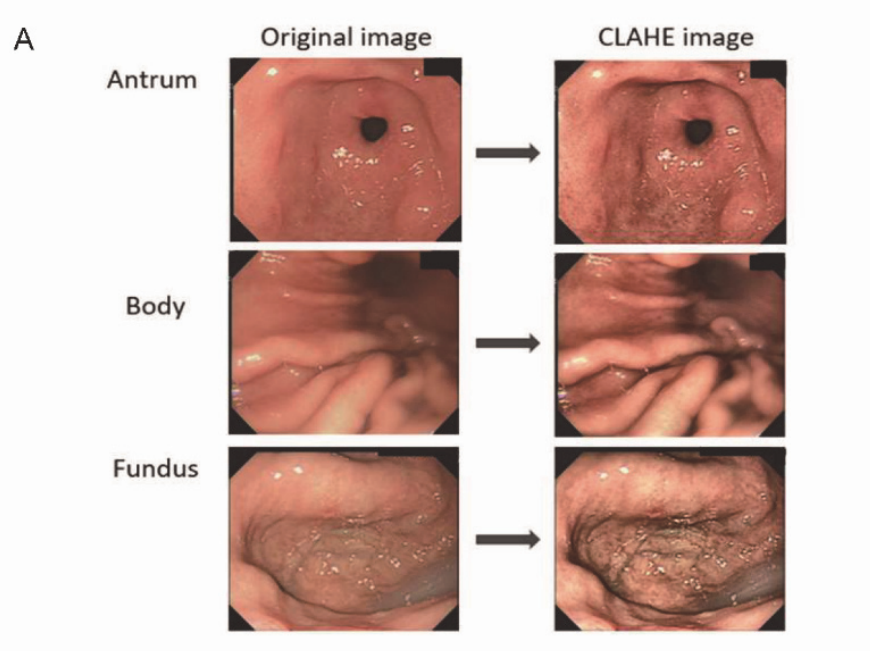

Fig. 10s contd.

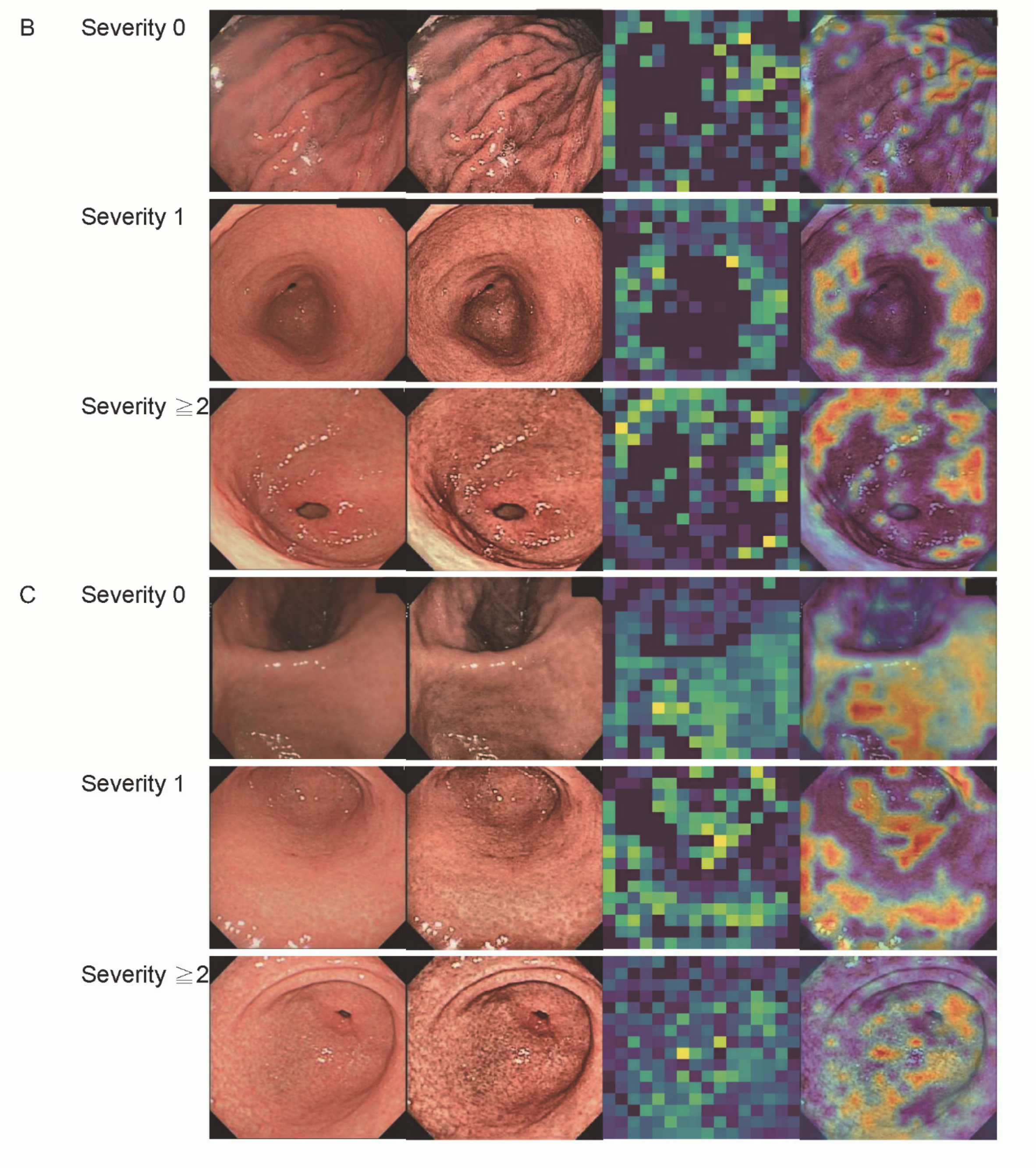

Fig. 11s Per-image basis interpretability analysis, as visualized through the mobile PACS.

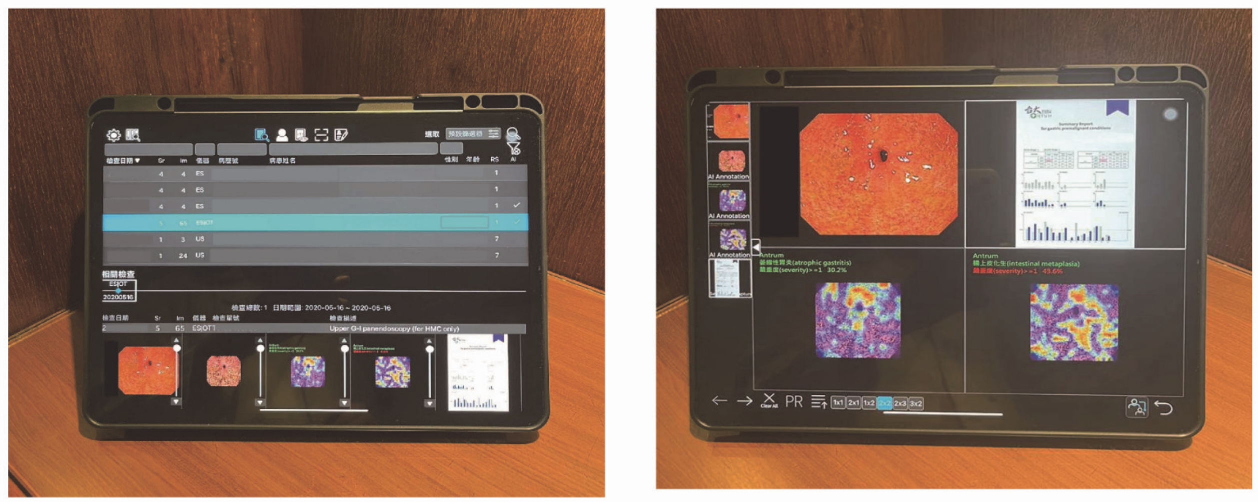

Fig. 12s Strategies and health status in the decision tree model.
Two strategies are compared: traditional endoscopic examination and AI-assisted interpretation. The square represents the decision node, where a strategy is selected. The purple encircled letter ‘M’ indicates the Markov node, with branches denoting the possible health states during each annual transition. The green circle represents the chance node, showing the probability of each event, while the red triangle represents the terminal node, marking the end of a pathway within a one-year cycle. Overall, the Markov model comprises nine distinct health states. Death is modeled as the absorbing state with no further transitions. Gastric cancer follows its treatment pathway, whereas *H. pylori*-positive, *H. pylori*-negative, post-endoscopic screening (*H. pylori* positive and negative), eradicated *H. pylori*, and premalignant conditions each progress along distinct pathways in subsequent cycles.

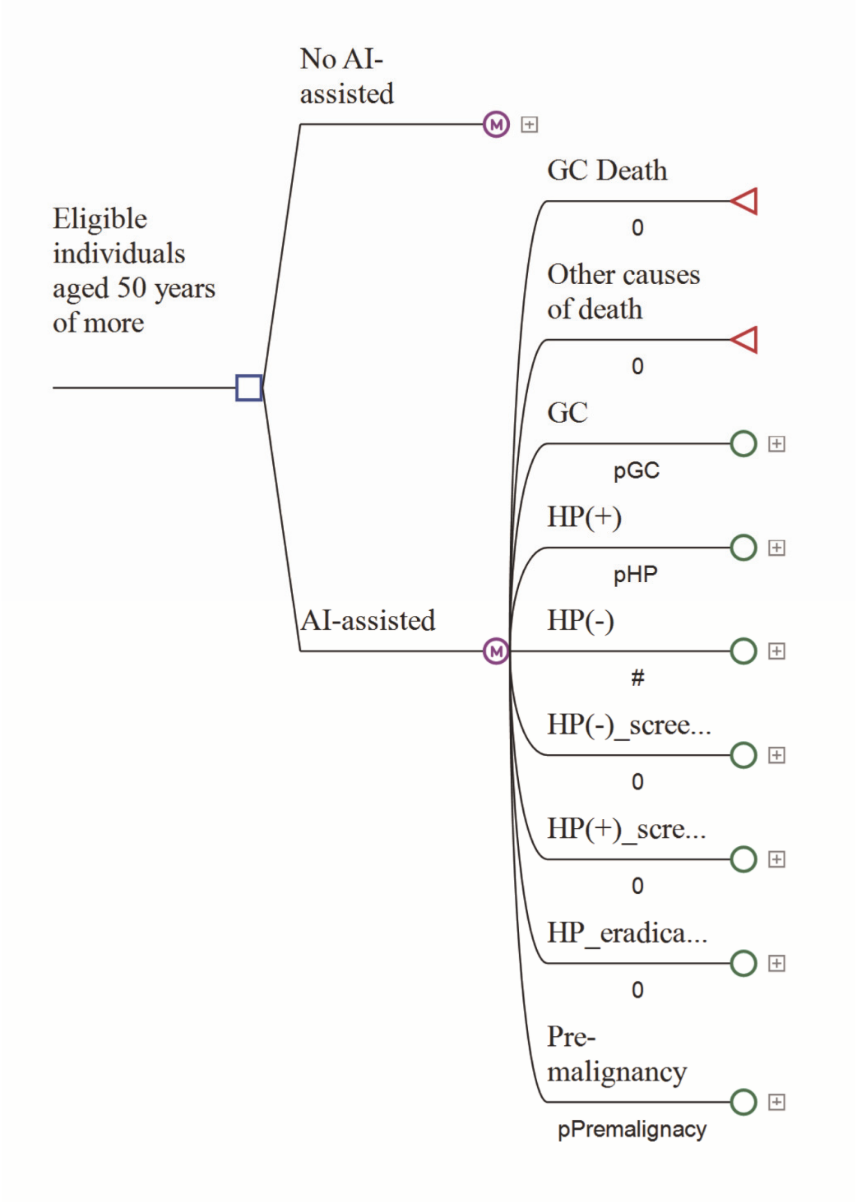

Fig. 13s Examples of pathways for individuals receiving AI-assisted interpretation in the cost-effectiveness model.
(A) For an individual with *H. pylori* infection, after accounting for the possibilities of death from other causes or the development of gastric cancer, the person may undergo endoscopic examination with AI-assisted interpretation. Depending on test sensitivity, the individual may then receive *H. pylori* testing and treatment, followed by a post-eradication course with a reduced risk of gastric cancer. For individuals without *H. pylori* infection, test specificity is applied in the pathway in a similar manner.
(B) For an individual with premalignant conditions, the person may likewise undergo endoscopy with AI-assisted interpretation. If the test result is positive, the individual will enter an endoscopic surveillance program, with a lower risk of progression to gastric cancer death due to early detection. For individuals without premalignant condition, test specificity is applied in the pathway.

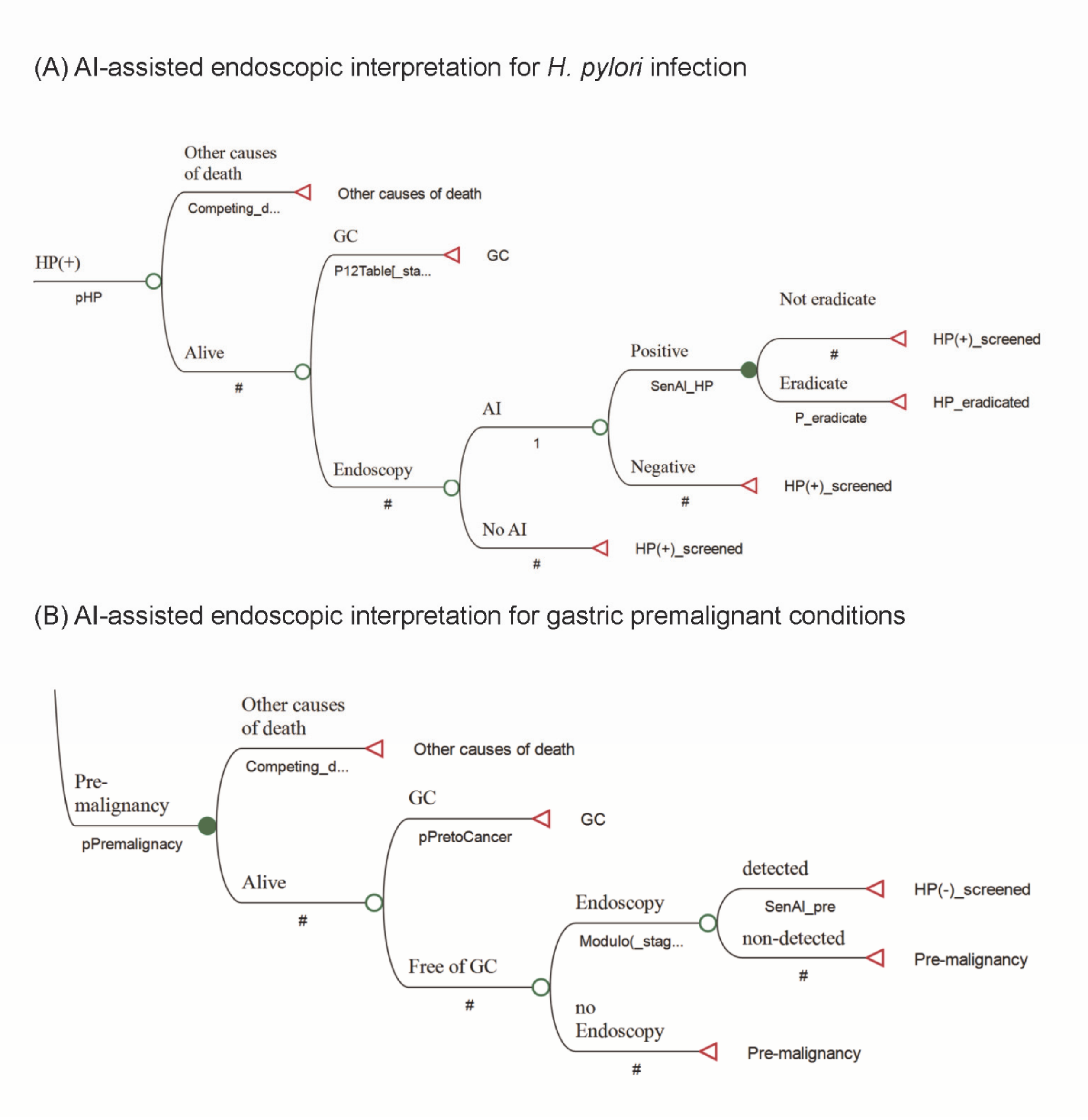

Fig. 14s The model selection process.
The performance of the AI models at each step were compared. The bar charts compare the accuracies for different models, with the error bars indicating the 95% confidence intervals. A: To exclude organic lesions and enhanced images; B: To classify the stomach from non-stomach images; C: To classify stomach images according to the anatomical locations; D: To classify the presence of premalignant gastric conditions; E: To classify the presence of *H. pylori* infection.

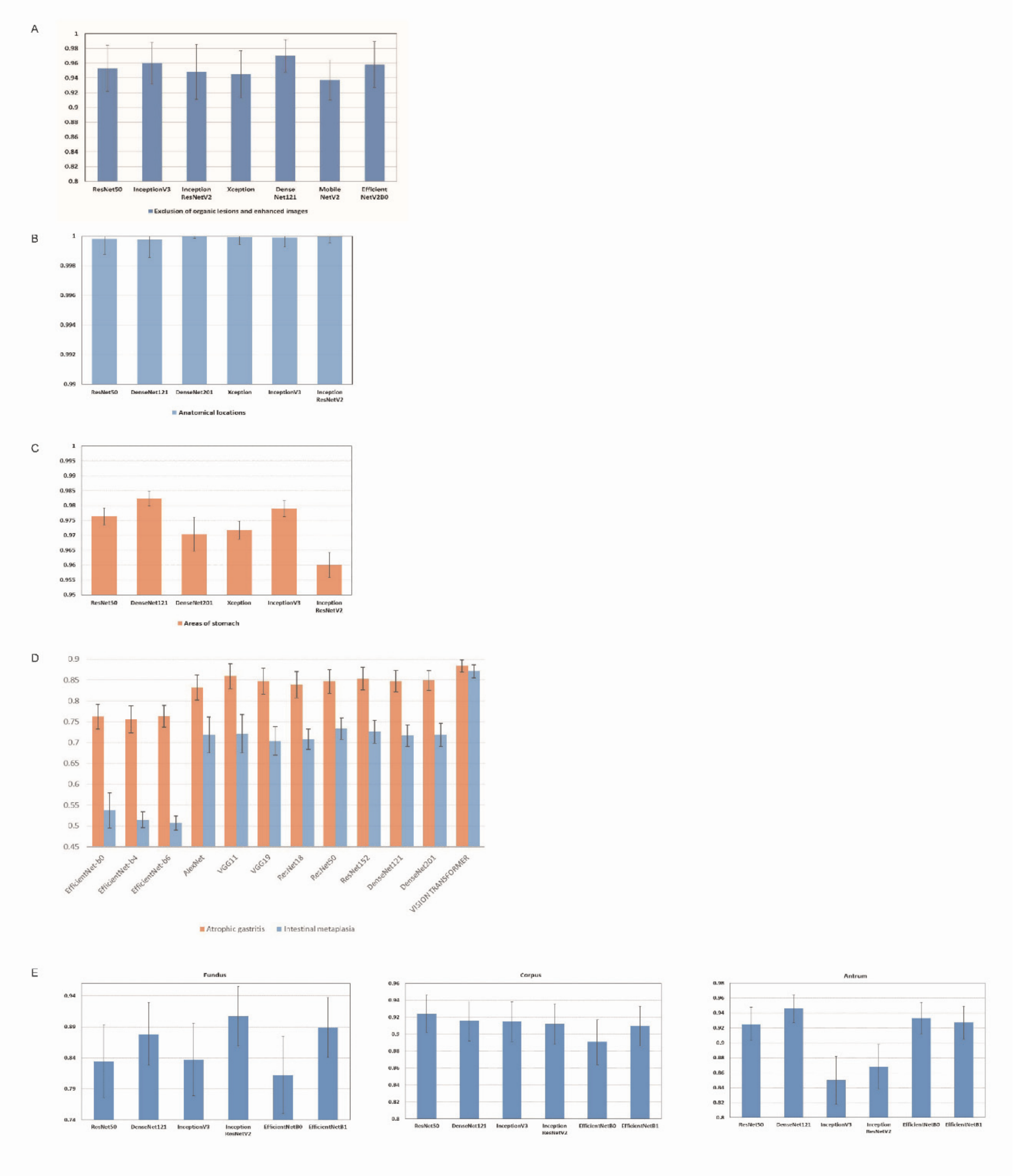

Fig. 15s The comprehensive interpretability analyses.
In the comprehensive interpretability analyses, the classification study employed two machine learning algorithms, including the logistic regression and Support Vector Machine (SVM). For hyperparameter tuning, the Bayesian Optimization was used to automatically search for the best hyperparameter combination. This method calculated the expected value and standard deviation of the data through Gaussian Process Regression. A larger expected value indicated a better set of hyperparameters, which was more likely to lead to better training results on real data. A larger standard deviation, on the other hand, indicated higher uncertainty or variability in the hyperparameters. Finally, the gastric cancer risk assessment model outputted a prediction probability value between 0 and 1, with a threshold of 0.538. If the value was less than 0.538, it outputs 0, indicating low risk for gastric cancer; if the value was greater than 0.538, it outputs 1, indicating high risk for gastric cancer. The logistic regression and SVM models had similar performance for the classification of gastric cancer risk (A). The ROC curve analyses (B) show the improved performance of the basic model, intermediate model, and full model in classifying patients with and without subsequent gastric cancer. The SHAP value on the x-axis represents the impact of a risk factor on the model's prediction, indicating how much it contributes to changing the prediction from the baseline. Active *H. pylori* infection, presence of intestinal metaplasia in the antrum, older age, presence of intestinal metaplasia in the body, and presence of atrophic gastritis in the body, in this order, have a stronger association with gastric cancer risk. A representative case of the comprehensive interpretability analyses was shown in C.

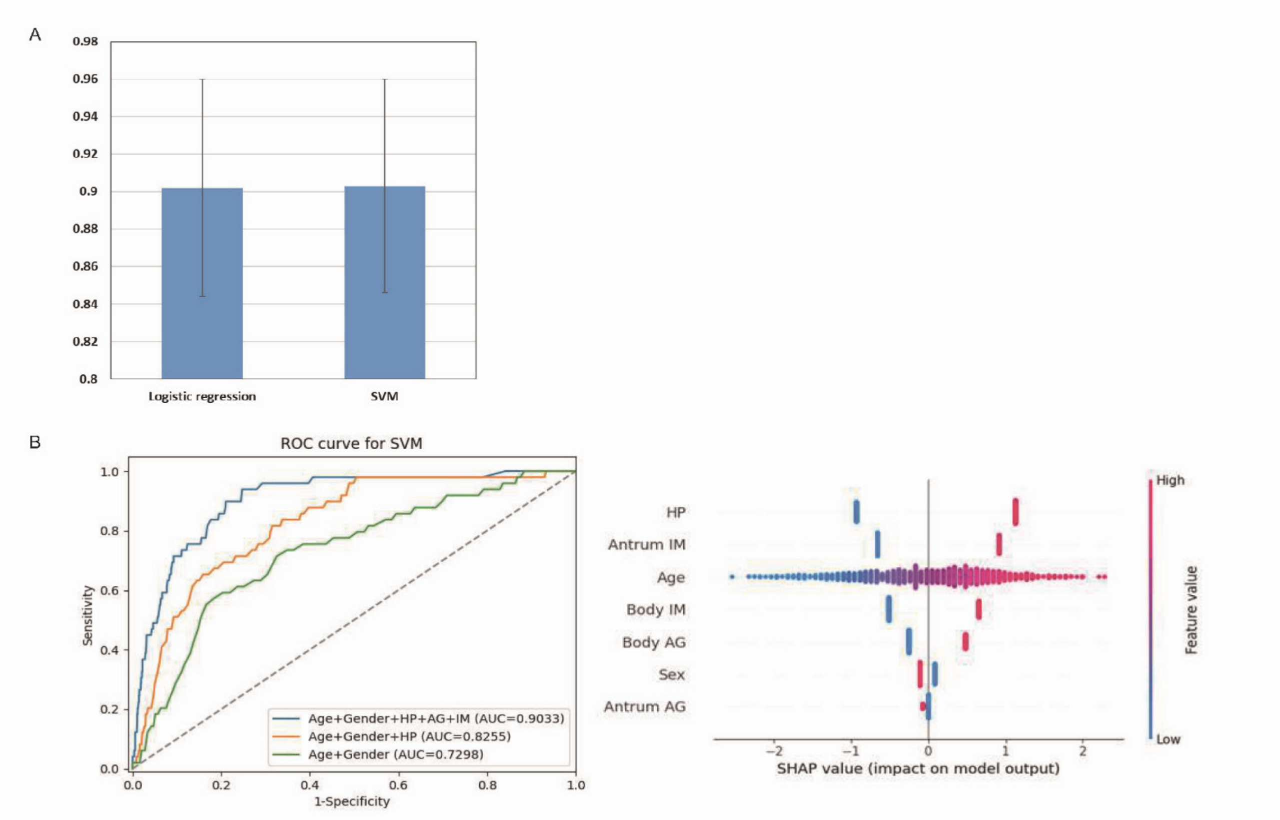

Figure 15c

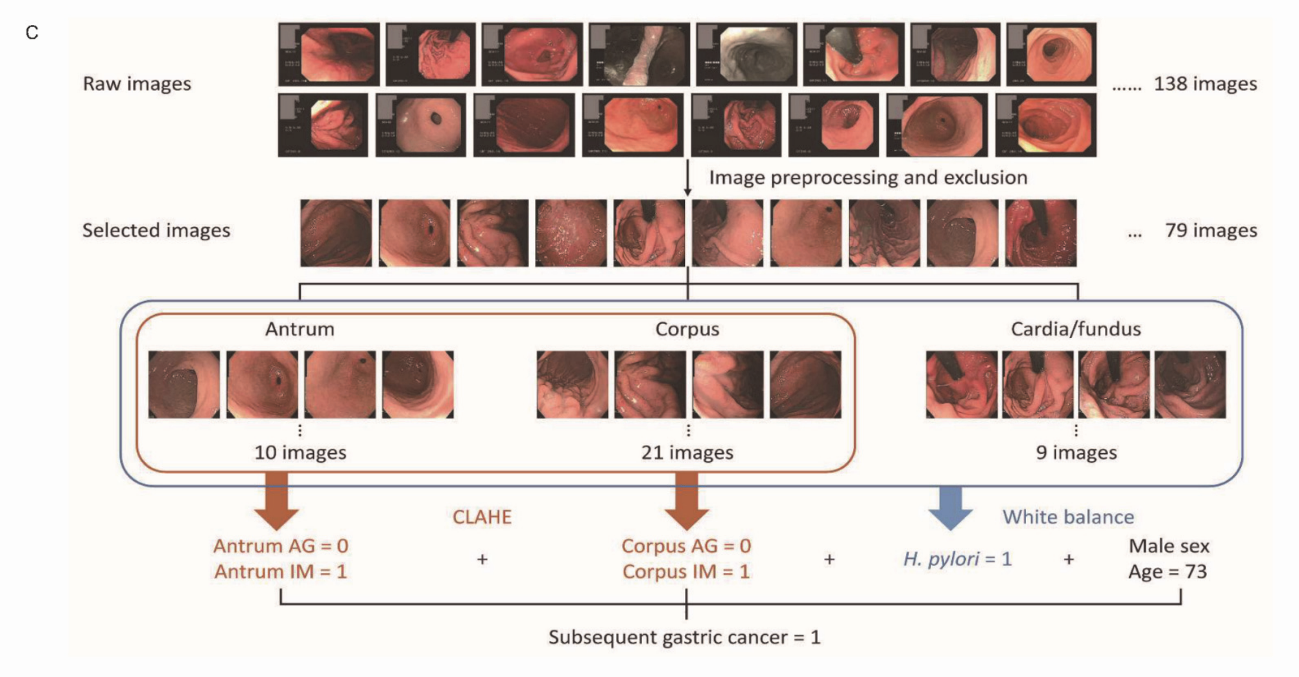
C
Raw images
...... 138 images
Image preprocessing and exclusion
Selected images
... 79 images
Antrum
10 images
Corpus
21 images
Cardia/fundus
9 images
CLAHE
White balance
Antrum AG = 0
Antrum IM = 1
+
Corpus AG = 0
Corpus IM = 1
+
H. pylori = 1
+
Male sex
Age = 73
Subsequent gastric cancer = 1

Fig. 16s The cost-effectiveness analysis results presented in terms of the incremental cost-effectiveness ratio per life-year gained.
The AI-assisted approach is associated with lower costs and greater effectiveness, resulting in a cost-saving strategy, compared with the traditional approach.

Cost-effectiveness plane

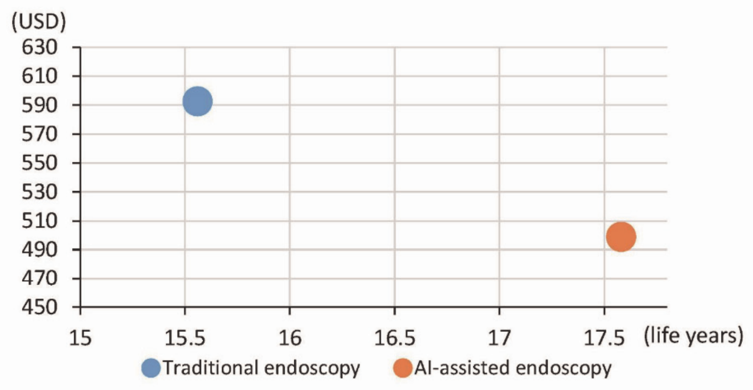

Incremental cost-effectiveness ratio

Strategy	Cost	Effectiveness	Incremental cost	Incremental effectiveness	ICER
Traditional endoscopy	592.61	15.57	-	-	-
AI-assisted endoscopy	498.95	17.56	-93.67	2.02	-46.44
